# Causality between herpes virus infections and allograft dysfunction after tissue and organ transplantation: a two-sample bidirectional Mendelian randomization study

**DOI:** 10.3389/fimmu.2024.1411771

**Published:** 2024-08-14

**Authors:** Xiaojuan Qiu, Tianjiao Ma, Shishun Zhao, Zongyu Zheng

**Affiliations:** ^1^ Department of Urology, The First Hospital of Jilin University, Changchun, China; ^2^ College of Mathematics, Jilin University, Changchun, China; ^3^ Department of Rheumatology and Immunology, China-Japan Union Hospital of the Jilin University, Changchun, China

**Keywords:** Mendelian randomization, tissue and organ transplantation, allograft dysfunction, herpes virus infection, antibody

## Abstract

**Background:**

Observational studies have suggested that herpes virus infections increase the risk of allograft dysfunction after tissue and organ transplantation, but it is still unclear whether this association is causal. The aim of this study was to assess the causal relationship between four herpes virus infections and allograft dysfunction.

**Methods:**

We used two-sample bidirectional Mendelian randomization (MR) to investigate the causality between four herpes virus infections — cytomegalovirus (CMV), Epstein-Barr virus (EBV), herpes simplex virus (HSV) and varicella zoster virus (VZV) — and allograft dysfunction after tissue and organ transplantation. Based on summary data extracted from genome-wide association studies (GWAS), we chose eligible single nucleotide polymorphisms (SNPs) as instrumental variables. The Inverse variance weighted (IVW) method was used as the main analysis method, supplemented by Weighted median and MR-Egger analyses. The MR-PRESSO test, MR-Egger intercept test, heterogeneity test, leave-one-out analysis and funnel plot were used to analyze the sensitivity of MR results.

**Results:**

We found EBV early antigen-D (EA-D) antibody levels and shingles were the only two variables associated with an increased risk of allograft dysfunction. No evidence of allograft dysfunction increasing the risk of the four herpes virus infections was observed. Sensitivity analyses confirmed the robustness of our results.

**Conclusions:**

Our results suggest that EBV and VZV are involved in graft rejection or dysfunction. However, the relationship between CMV and HSV infections and allograft dysfunction remains unclear and requires further clarification.

## 1 Introduction

Solid organ transplantation (SOT) has been an established and practical definitive treatment option for patients with end-organ dysfunction, and has transformed the survival and quality of life of patients with end-organ dysfunction (1). However, allograft dysfunction can affect the survival of grafts and SOT recipients. In this study, allograft dysfunction was defined as failure and rejection of transplanted organs and tissues due to external causes. Although there are many external factors that can cause allograft dysfunction, infectious diseases after SOT are a significant cause of chronic allograft dysfunction and allograft Survival (2).

Herpes virus is a common opportunistic virus after transplantation. These DNA viruses are divided into four subfamilies based on their physicochemical properties: (i) α herpes viruses such as herpes simplex virus (HSV) or varicella zoster virus (VZV), (ii) β herpes viruses such as cytomegalovirus (CMV), (iii) γ herpes viruses such as Epstein-Barr virus (EBV), and (iv) unclassified herpes viruses (3). In Europe, the infection rate of herpes viruses in the general population is as high as 95% for HSV and VZV, 90% for EBV, and 60% for CMV (4), with prevalence rate increasing with age (4). Due to the administration of immunosuppressants, organ transplant recipients generally have weakened immunity. Consequently, the incidence of postoperative secondary herpes virus infection is significantly higher, increasing the risk of disease and mortality among this population (5–9).

Previous studies have shown that CMV is the primary cause of infectious diseases within the first year following solid organ transplantation (SOT), and CMV is also considered a risk factor for allograft dysfunction and rejection (10). Similarly, post-transplant lymphoproliferative disorders resulting from EBV infection are considered as one of the most severe complications of organ transplantation, often occurring in the early post-transplant period (11, 12). The mortality rate among transplant recipients suffering from post-transplant lymphoproliferative disorders has been reported to be as high as 60% (13). Furthermore, up to 70% of SOT recipients may develop VZV or HSV infections if preventive measures are not taken, some of which can be life-threatening and pose a risk to the transplanted organ (14). VZV and two HSV have also been reported to establish a lifelong latency period in the ganglia of SOT patients after the initial primary infection (14). Therefore, after tissue and organ transplantation, the use of antiviral drugs or the addition of immunoglobulin to suppress herpes virus infection has become a widespread consensus (15–17).

While there is scientific evidence supporting that CMV, EBV, VZV and HSV increase the risk of rejection or death after tissue and organ transplants (10–14), there is currently no direct evidence of a causal relationship. In fact, many of the observational studies performed in this field presented numerous shortcomings, such as residual and unmeasured confounding, detection bias, and reverse causality (18, 19). In recent years, Mendelian randomization (MR) has emerged as a powerful technique for inferencing causality based on genome-wide association studies (GWAS) (19, 20).

MR uses genetic variation as an instrumental variable (IV) to infer whether a risk factor has a causal effect on outcomes (20, 21). In MR studies, genetic variation follows the principle of assigning random alleles to offspring, similar to randomized controlled trials (22). This approach effectively mitigates the confounding factors and reverse causality that are often encountered in observational studies (23). MR has been widely applied in herpes virus research. For instance, MR studies have shown that there is no causal relationship between herpes virus infection and pulmonary fibrosis (24), that CMV infection dose not significantly increase the risk of autism spectrum disorder (25), or that there is a causal relationship between EBV infection and Alzheimer’s disease (26). Recent MR studies have also shown that lipids may trigger causal pathological processes that lead to allograft dysfunction after organ and tissue transplantation (27). However, to our knowledge, there are no studies investigating a potential causal relationship between herpes virus infections and tissue and organ transplant dysfunction.

Herein, we used a two-sample bidirectional MR to assess the causal relationship between four herpes virus (CMV, EBV, HSV, VZV) infectious diseases, associated antibody and immunoglobulin G (IgG) levels, and allograft dysfunction after tissue and organ transplantation.

## 2 Methods

### 2.1 Study design

**Figure 1:**
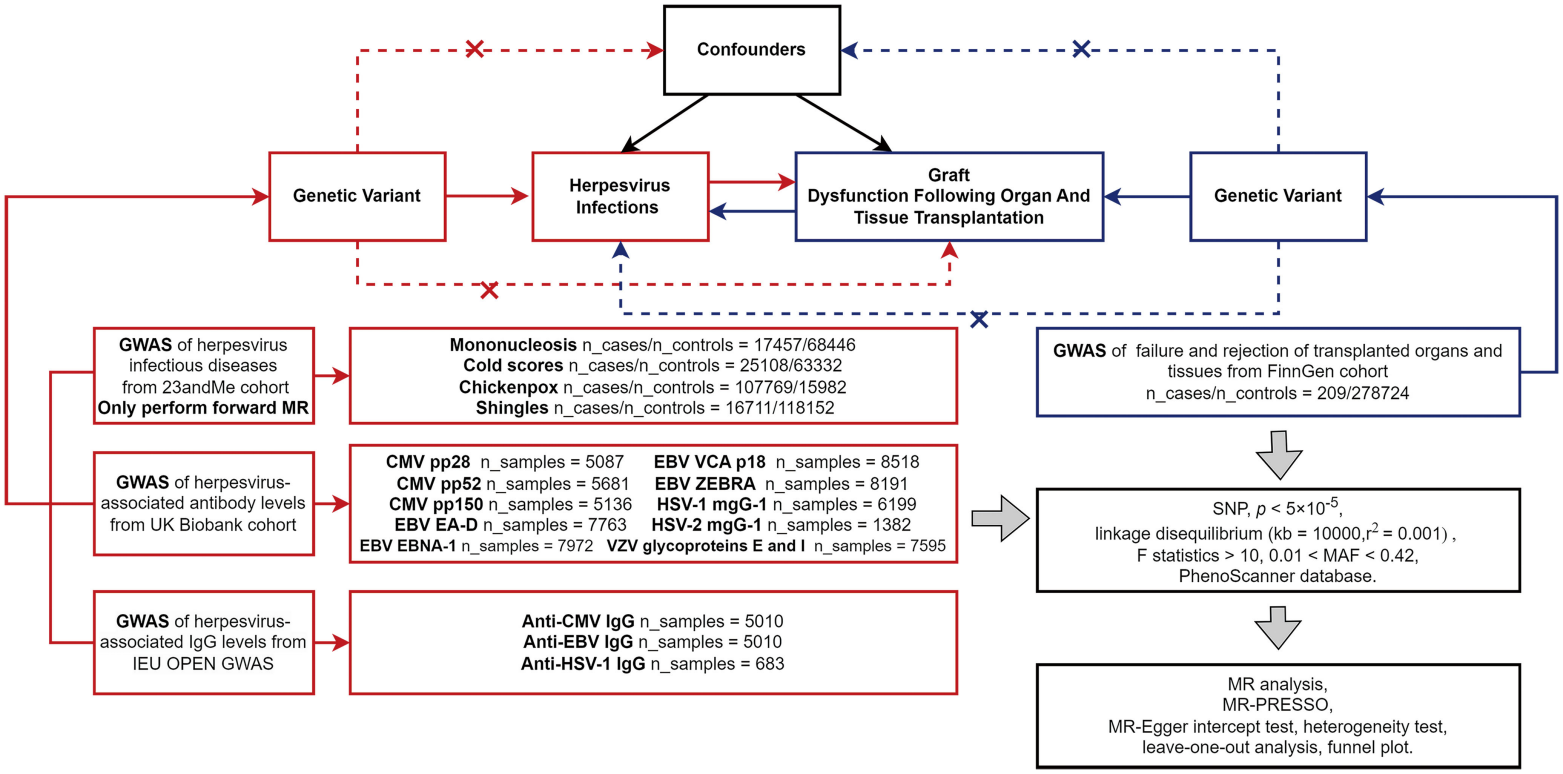
The flow chart of MR Design. In the forward MR analysis, exposures (herpes virus infections) are shown in red, and outcome (allograft dysfunction after organ and tissue transplantation) is shown in blue; In the reverse MR Analysis, exposure (allograft dysfunction) is shown in blue, and outcomes (herpes virus infections) are shown in red. Abbreviation: GWAS, genome-wide association study; MR-PRESSO, MR pleiotropy residual sum and outliers; MR, Mendelian randomization; SNP, single nucleotide polymorphisms.

MR Studies need to meet the following assumptions: First, IVs should be closely related to exposure; Second, IVs are not associated with any possible confounders; Third, IVs can only affect the outcome through exposure (20). When an IV can affect the outcome through a path other than genetic variant-expose-outcome, we consider the IV to have horizontal pleiotropy. The data in this study came from publicly available GWAS databases (**Table 1**; **Supplementary Table 1**). All consortiums initially involved in the GWAS studies completed the participants’ ethical approval and written informed consent. **Figure 1** summarizes the flow chart of a two-sample bidirectional MR Design.

**Table 1:** Brief description of datasets utilized in the Mendelian randomization study.

Phenotype	GWAS ID	Source	Sample size (Cases\Controls)	Population
Mononucleosis	mononucleosis	23andMe cohort	17457\68446	European
Cold scores	cold scores		25108\63332	European
Chickenpox	chickenpox		107769\15982	European
Shingles	shingles		16711\118152	European
CMV pp28 antibody levels	ebi-a-GCST90006894	UK Biobank cohort	5,087	European
CMV pp52 antibody levels	ebi-a-GCST90006895		5,681	European
CMV pp150 antibody levels	ebi-a-GCST90006896		5,136	European
EBV EA-D antibody levels	ebi-a-GCST90006898		7,763	European
EBV EBNA-1 antibody levels	ebi-a-GCST90006899		7,972	European
EBV VCA p18 antibody levels	ebi-a-GCST90006900		8,518	European
EBV ZEBRA antibody levels	ebi-a-GCST90006901		8,191	European
HSV-1 mgG-1 antibody levels	ebi-a-GCST90006918		6,199	European
HSV-2 mgG-1 antibody levels	ebi-a-GCST90006920		1,382	European
VZV glycoproteins E and I antibody levels	ebi-a-GCST90006929		7,595	European
Anti-CMV IgG levels	ieu-b-4900	IEU OPEN GWAS	5,010	European
Anti-EBV IgG levels	ieu-b-4901		5,010	European
Anti-HSV-1 IgG levels	ieu-b-4906		683	European
Failure and rejection of transplanted organs and tissues	FAILU_REJEC_TP_ ORGANS_TISSU	FinnGen cohort	209\278724	European

EBV, Epstein-Barr virus; CMV, cytomegalovirus; HSV, herpes simplex; VZV, Varicella zoster virus; EA, EBV early antigen; EBNA-1, EBV nuclear antigen-1; VCA, viral capsid antigen; IgG, immunoglobulin G.

### 2.2 GWAS data collection

Genomic data associated with herpesvirus infectious diseases was extracted from a previous GWAS study (28), that used the summary data of 23andMe cohort (only the top 8,000 SNPs are listed). Only participants of European ancestry >97% were included in the analysis (25, 28), and a rigorous self-report questionnaire on infection history was used to determine the phenotype. Specifically, we selected mononucleosis (17,457 cases and 68,446 controls) and cold sores (25,108 cases and 63,332 controls) caused by EBV and HSV, and chickenpox (107,769 cases and 15,982 controls) and shingles (16,711 cases and 118,152 controls) caused by HSV (**Table 1**). Since only the first 8,000 SNPS with the lowest *p*-value in the 23andMe cohort were available, the data were not used as exposure data for the reverse MR Study of allograft dysfunction and herpes virus infection. We obtained GWAS summary data related to herpesvirus-associated IgG levels from the IEU Open GWAS project (29, 30). We selected the GWAS summary data sets ieu-b-4900 (n = 5,010) for the study of anti-CMV IgG levels, ieu-b-4901 (n = 5,010) for investigating anti-EBV IgG levels and ieu-b-4906 (n = 683) for anti-HSV-1 IgG levels (**Table 1**). GWAS summary data on herpesvirus-associated antibody levels was collected from the UK Biobank cohort (31). We selected genomic data regarding antibody levels against CMV pp28 (n = 5,087), CMV pp52 (n = 5,681), CMV pp150 (n = 5,136), EBV early antigen-D (EA-D, n = 7,763), EBV nuclear antigen-1 (EBNA-1, n = 7,972), EBV viral capsid antigen (VCA) p18 (n = 8,518), EBV ZEBRA (n = 8,191), HSV-1 mgG-1 (n = 6,199), HSV-2 mgG-1 (n = 1,382), and VZV glycoprotein E and I (n = 7,595). We selected GWAS summary data for failure and rejection of transplanted organs and tissues that was described as injury, poisoning and certain other consequences of external causes (FAILU_REJEC_TRANSPLANTED_ORGANS_TISSU, 209 cases, 278,724 controls) from the FinnGen cohort (32).

The study used the large publicly available GWAS databases, which have received approval from their relevant ethical review board and participants.

### 2.3 Instrumental variable identification

Consistent with previous studies (27, 33), to obtain a sufficient number of single nucleotide polymorphisms (SNPs), we chose a relatively loose threshold (*p*<5×10^-5^) for analysis. To ensure the selection of independent SNPs and minimize the influence of linkage disequilibrium (LD) on the results, SNPs were selected at a threshold of LD *r* ^2^>0.001 and a distance of 10,000 kb (34). The strength of the correlation between the instrumental variable and the exposure factor was assessed by the F-statistic. To mitigate the bias caused by weak instrumental variables, we only consider SNPs with F-statistics >10 (35, 36). We excluded SNPs with a minor allele frequency (MAF) of less than 0.01 because the effects of these SNPs were observed not to be stable (24), and deleted palindromic sequences with minor allele frequency (MAF>0.42) to prevent chain ambiguity errors (37). In addition, since a pleiotropic effect between lipids and allograft dysfunction was observed in the original GWAS study (27), We searched the PhenoScanner website (38–40) to exclude SNPs associated with blood lipids (high-density lipoprotein, low-density lipoprotein, cholesterol, and triglycerides) in the relationship between herpes virus and allograft dysfunction. These SNPs were genome-wide significant *(p<*5×10^-5^) and known as confounding factors (**Supplementary Table 2**) (24).

### 2.4 Statistical analysis

We conducted a two-sample bidirectional Mendelian randomization study using the “TwoSampleMR” package (version 0.5.8) (41) in R software (version 4.2.1) (42) to investigate the relationship between four herpes viruses and allograft dysfunction after tissue and organ transplantation.

We mainly used Inverse variance weighting (IVW), the weighted median and MR-Egger method to carry out MR analysis to obtain the odds ratio (OR) estimates and *p*-values of causal effect, in which IVW method was used as the main method. When *p*< 0.05, the causal relationship between exposure and outcome was considered significant. In fixed effects meta-analyses, SNP-exposure coefficients and SNP-outcome coefficients were combined using IVW methods to give an overall estimate of causal effects (43). This is equivalent to a weighted regression of the SNP-outcome coefficient to the SNP-exposure coefficient with a zero intercept. The causal estimate for the IVW analysis represents a causal increase in outcome per unit change in exposure. The method assumes that all variables are valid IVs based on the MR assumption (**Figure 1**) and have no horizontal pleiotropy. To account for potential violations of the assumptions underlying the IVW MR analysis, we compared the IVW results with the Weighted median and MR-Egger methods, known to be more robust for horizontal pleiotropy, albeit at the cost of reduced statistical power (44). First, we employed the Weighted median MR method that allows 50% of the instrumental variables to be invalid (45). Secondly, we used MR-Egger regression based on the “NO Measurement Error” (NOME) assumption. This method allows all instrumental variables to be affected by horizontal pleiotropy, intercept represents the causal estimation deviation due to pleiotropy, and slope represents the causal estimation effect (46). Therefore, the MR-Egger regression intercept can assess the pleiotropy and provide an estimation effect that is not affected by pleiotropy. In addition to the MR-Egger regression intercept, MR pleiotropy residual sum and outliers (MR-PRESSO) tests are also used to detect outliers and horizontal pleiotropy (47). A *p*> 0.05 indicated no significant horizontal pleiotropy.

Since the exposure and outcome of two-sample MR came from different samples, there could be different population heterogeneity. We used the Cochran’s s Q statistic (IVW method) and Rucker’s s Q statistic (MR-Egger method) for heterogeneity tests (47). A *p*> 0.05 indicated no significant heterogeneity. The funnel plots were also used to assess for heterogeneity among individual genetic variants. When there was no heterogeneity, the funnel plot was symmetrical. In addition, a “leave-one-out” analysis was performed to examine whether the causal relationship between exposure and outcome was influenced by a single SNP by removing SNPs one by one to see whether the OR changes significantly (48). The MR results were visualized using forest plots and scatter plots (“TwoSampleMR” package). The forest plots present the estimated causal effect for each SNP. Each point in the scatter plots represents a SNP, showing how each genetic variation is associated with exposure and outcome.

## 3 Results

The results of MR-PRESSO, pleiotropy test and heterogeneity test are shown in **Supplementary Table 3**. Scatter plots, leave-one-out plots, forest plots and funnel plots of MR Analysis results are shown in **Supplementary Materials** (**Supplementary Figures 1-30**).

### 3.1 Effect of CMV infection on allograft dysfunction

**Figure 2:**
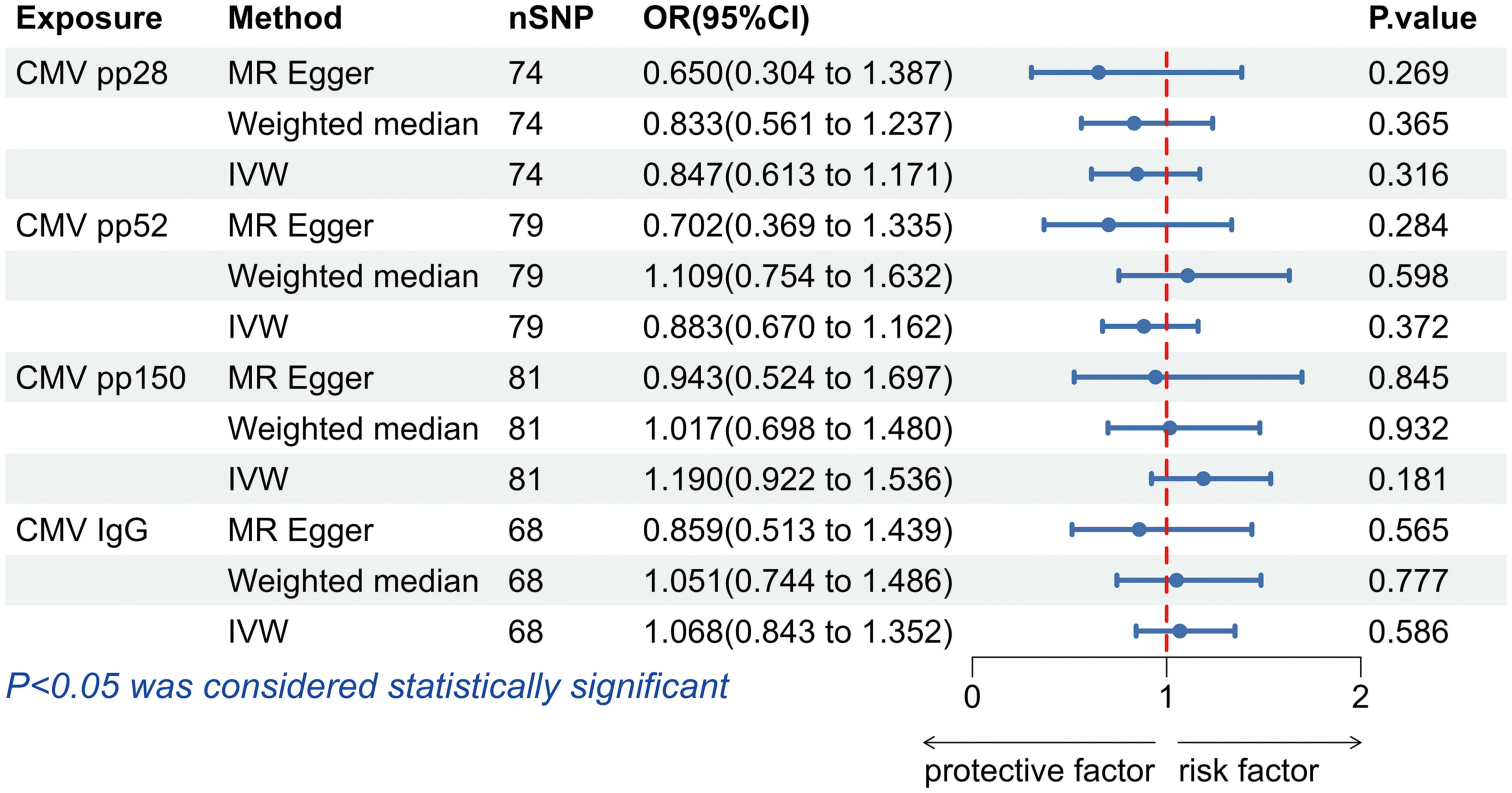
The forest plot of the causal relationship between cytomegalovirus and allograft dysfunction. CMV, cytomegalovirus; nSNP, number of single nucleotide polymorphisms; OR, odds ratio; CI, confidence interval; IVW, inverse variance weighted.

IVW results did not support that antibody levels against CMV pp28 (OR = 0.847, 95% confidence interval (CI): 0.613-1.171, *p* = 0.316),CMV pp52 (OR = 0.883, 95% CI: 0.670-1.162, *p* = 0.372), CMV pp150 (OR = 1.190, 95% CI: 0.922-1.536, *p* = 0.181) and anti-CMV IgG (OR = 1.068, 95% CI: 0.843-1.352, *p* = 0.586) had effects on allograft dysfunction (**Figure 2**). Similarly, the results obtained using the Weighted median and MR-Egger methods did not support a causal relationship between CMV infection and allograft dysfunction either (**Figure 2**).

### 3.2 Effect of EBV infection on allograft dysfunction

**Figure 3:**
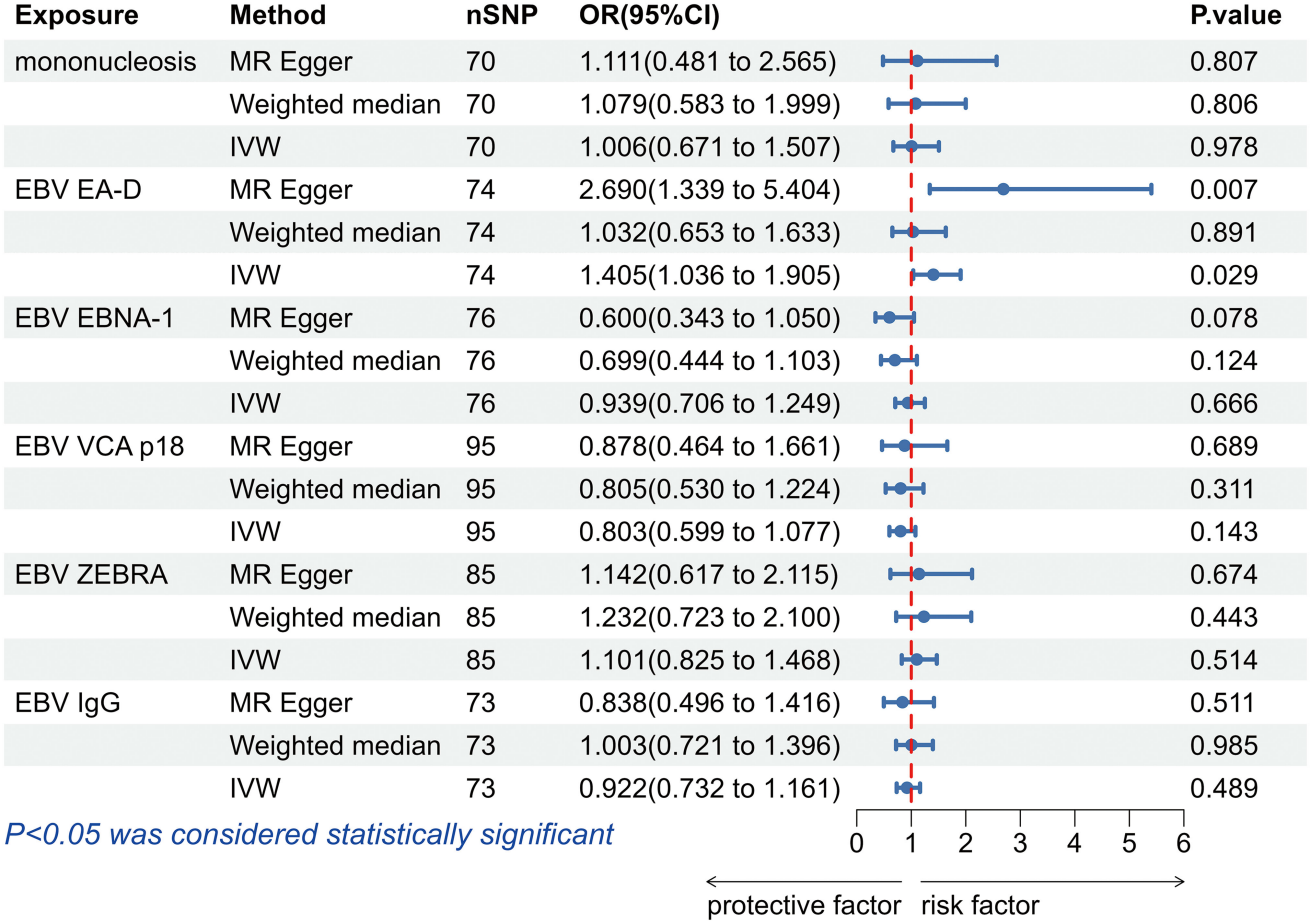
The forest plot of the causal relationship between Epstein-Barr virus and allograft dysfunction. EBV, Epstein-Barr virus; nSNP, number of single nucleotide polymorphisms; OR, odds ratio; CI, confidence interval; IVW, inverse variance weighted.

**Figure 4:**
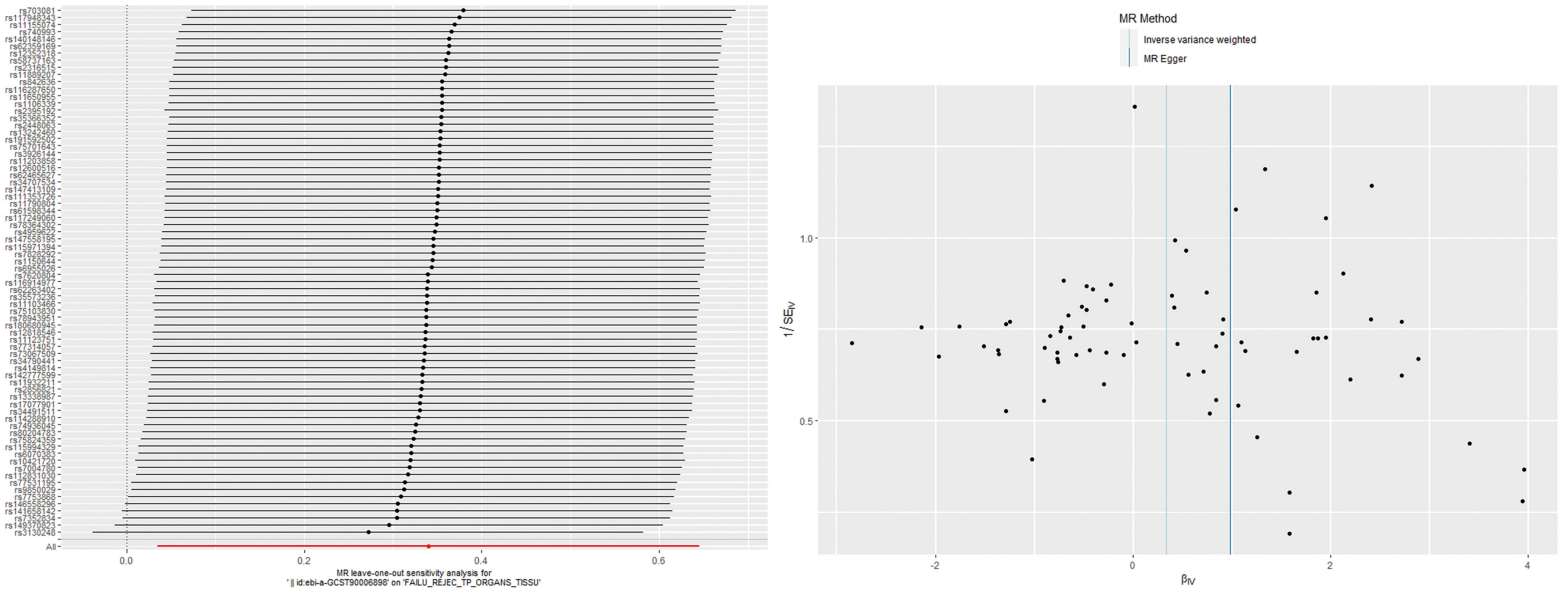
The leave-one-out plot and funnel plot of EBV EA-D antibody levels and allograft dysfunction.

The IVW analysis found a positive effect of EBV EA-D antibody levels on allograft dysfunction (OR = 1.405, 95% CI:1.036-1.905, *p* = 0.029). And the OR greater than 1 indicated that higher antibody levels would increase the risk of allograft dysfunction. There was no other evidence of a causal relationship between the other EBV antibody levels, mononucleosis and EBV IgG levels, and allograft dysfunction (**Figure 3**). However, the calculated *p*-value of Egger intercept for EBV EA-D antibody levels was 0.046, indicating that there is some evidence of directional horizontal pleiotropy in the MR analysis, and therefore a potential bias in the causal estimate derived from the MR analysis (**Table 2**). Under this circumstance, we used the MR-Egger method to provide a more reliable estimate (49, 50), and it still indicated a causal relationship between EBV EA-D antibodies and allograft dysfunction (OR = 2.690, 95% CI: 1.339-5.404, *p* = 0.007). No heterogeneity was found with the Cochran’s Q and Rucker’s Q tests for EBV EA-D antibody levels (*p* = 0.533, *p* = 0.644) (**Table 2**). Moreover, the leave-one-out plot of EBV EA-D antibody levels showed that the sequential removal of each SNP had little effect on the results, and no single SNP had a significant effect on the overall causal effect estimate. The funnel plot is essentially symmetrical, indicating the robustness of this result (**Figure 4**).

**Table 2:** The pleiotropic and heterogeneous results of EBV EA-D antibody levels and allograft dysfunction.

Exposure	Outcome	MR-PRESSO		Pleiotropy test	Heterogeneity test	
		Distortion test	Global test	Egger intercept	Cochran’s Q test	Rucker’s Q test
					P-value	P-value
		Outliers	P-value	P-value	IVW	MR-Egger
EBV EA-D	FAILU_REJEC_TP_ORGANS_TISSU	NA	0.527	0.046	0.533	0.644

### 3.3 Effect of HSV infection on allograft dysfunction

**Figure 5:**
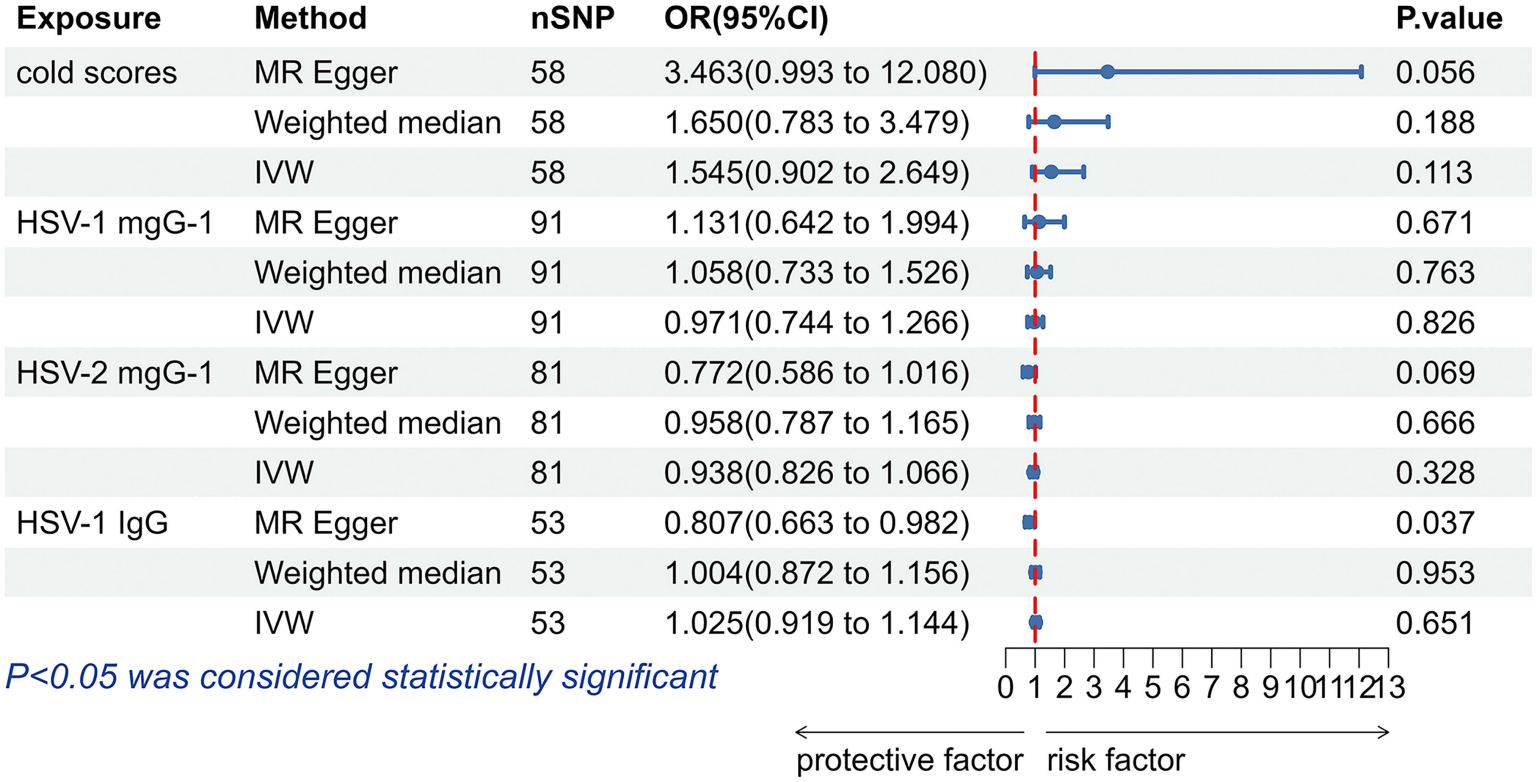
The forest plot of the causal relationship between herpes simplex virus and allograft dysfunction. HSV, herpes simplex virus; nSNP, number of single nucleotide polymorphisms; OR, odds ratio; CI, confidence interval; IVW, inverse variance weighted.

The results obtained with the IVW method did not support that antibody levels targeting HSV-1 mgG-1 (OR = 0.971, 95% CI: 0.744-1.266, *p* = 0.826), HSV-2 mgG-1 (OR = 0.938, 95% CI: 0.826-1.066, *p* = 0.328) and Anti-HSV-1 IgG (OR = 1.025, 95% CI: 0.919-1.144, *p* = 0.651), nor cold scores (OR = 1.545, 95% CI: 0.902-2.649, *p* = 0.113) had effects on allograft dysfunction (**Figure 5**). Likewise, the analyses performed using the Weighted median and MR-Egger methods did not support a causal relationship between HSV infection and allograft dysfunction either (**Figure 5**).

### 3.4 Effect of VZV infection on allograft dysfunction

**Figure 6:**
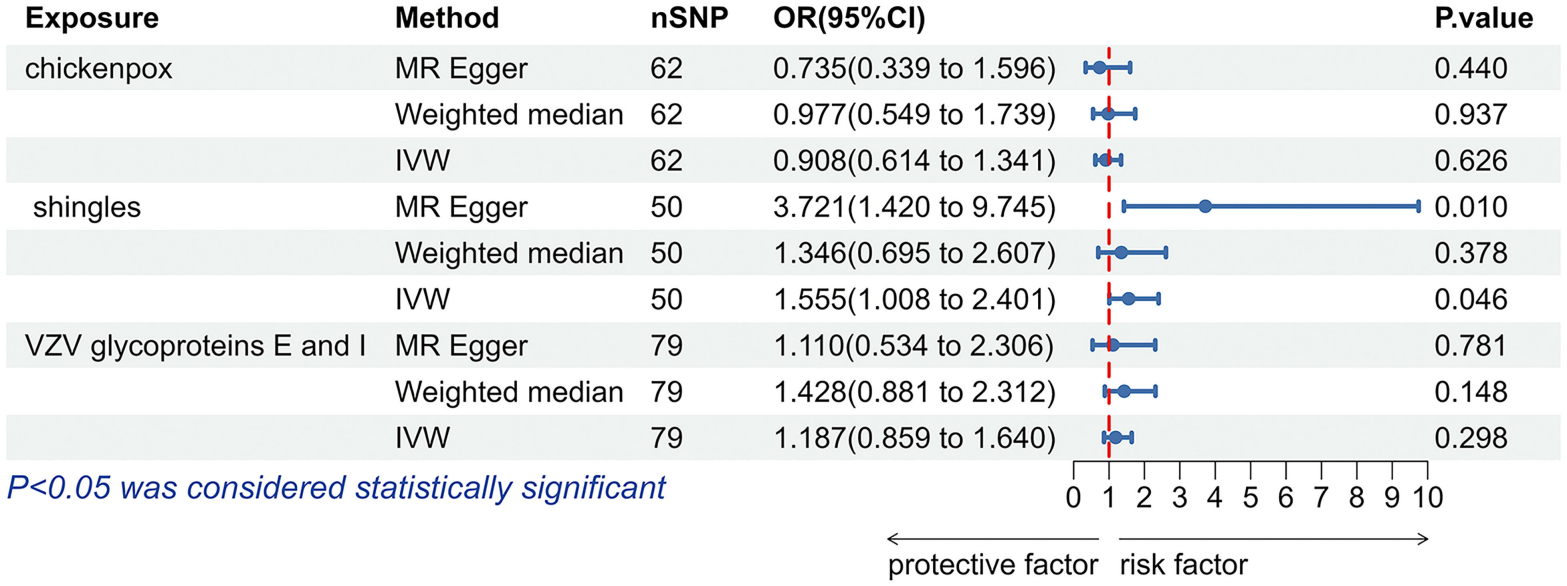
The forest plot of the causal relationship between varicella zoster virus and allograft dysfunction. VZV, varicella zoster virus; nSNP, number of single nucleotide polymorphisms; OR, odds ratio; CI, confidence interval; IVW, inverse variance weighted.

**Figure 7:**
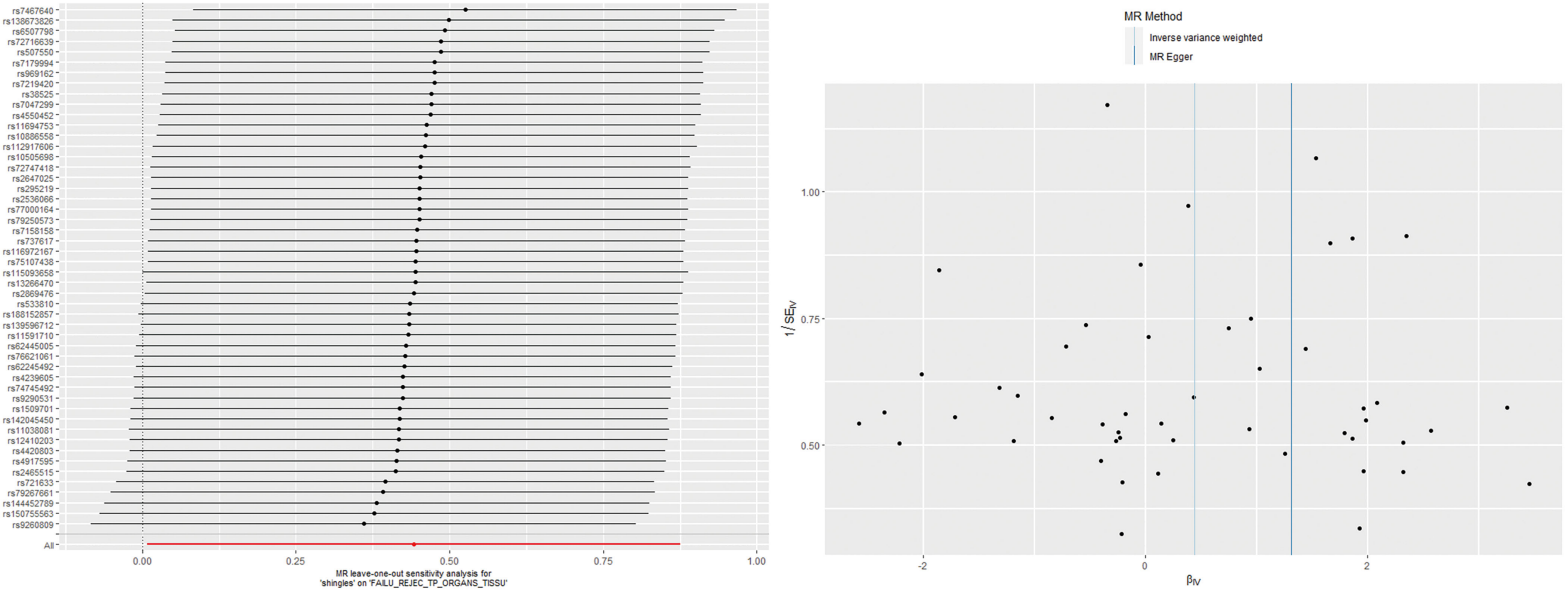
The leave-one-out plot and funnel plot of shingles and allograft dysfunction.

According to the IVW analysis results, shingles was positively associated with allograft dysfunction (OR = 1.555, 95% CI: 1.008-2.401, *p* = 0.046). On the contrary, there was no evidence of a causal relationship between chickenpox (OR = 0.908, 95% CI: 0.614-1.341, *p* = 0.626) and VZV glycoprotein E and I antibody levels (OR = 1.187, 95% CI: 0.859-1.640, *p* = 0.298), and allograft dysfunction (**Figure 6**). The MR-Egger method for shingles also confirmed this conclusion (OR = 3.721, 95%CI: 1.420-9.745, *p* = 0.010). Additionally, neither Cochran’s Q test nor Rucker’s Q showed heterogeneity in shingles (*p* = 0.792, *p* = 0.880) (**Table 3**). In addition, no significant MR-Egger intercept was observed (*p* = 0.052), and the MR-PRESSO test was not significant (*p* = 0.807), indicating no horizontal pleiotropy (**Table 3**). Furthermore, the leave-one-out analysis demonstrated the robustness of our MR Analysis, as it is not affected by any single SNP, and the funnel plot is nearly symmetrical (**Figure 7**).

**Table 3:** The pleiotropic and heterogeneous results of shingles and allograft dysfunction.

Exposure	Outcome	MR-PRESSO		Pleiotropy test	Heterogeneity test	
		Distortion test	Global test	Egger intercept	Cochran’s Q test	Rucker’s Q test
					P-value	P-value
		Outliers	P-value	P-value	IVW	MR-Egger
Shingles	FAILU_REJEC_TP_ORGANS_TISSU	NA	0.807	0.052	0.792	0.880

### 3.5 Effect of allograft dysfunction on herpes virus infection

**Figure 8:**
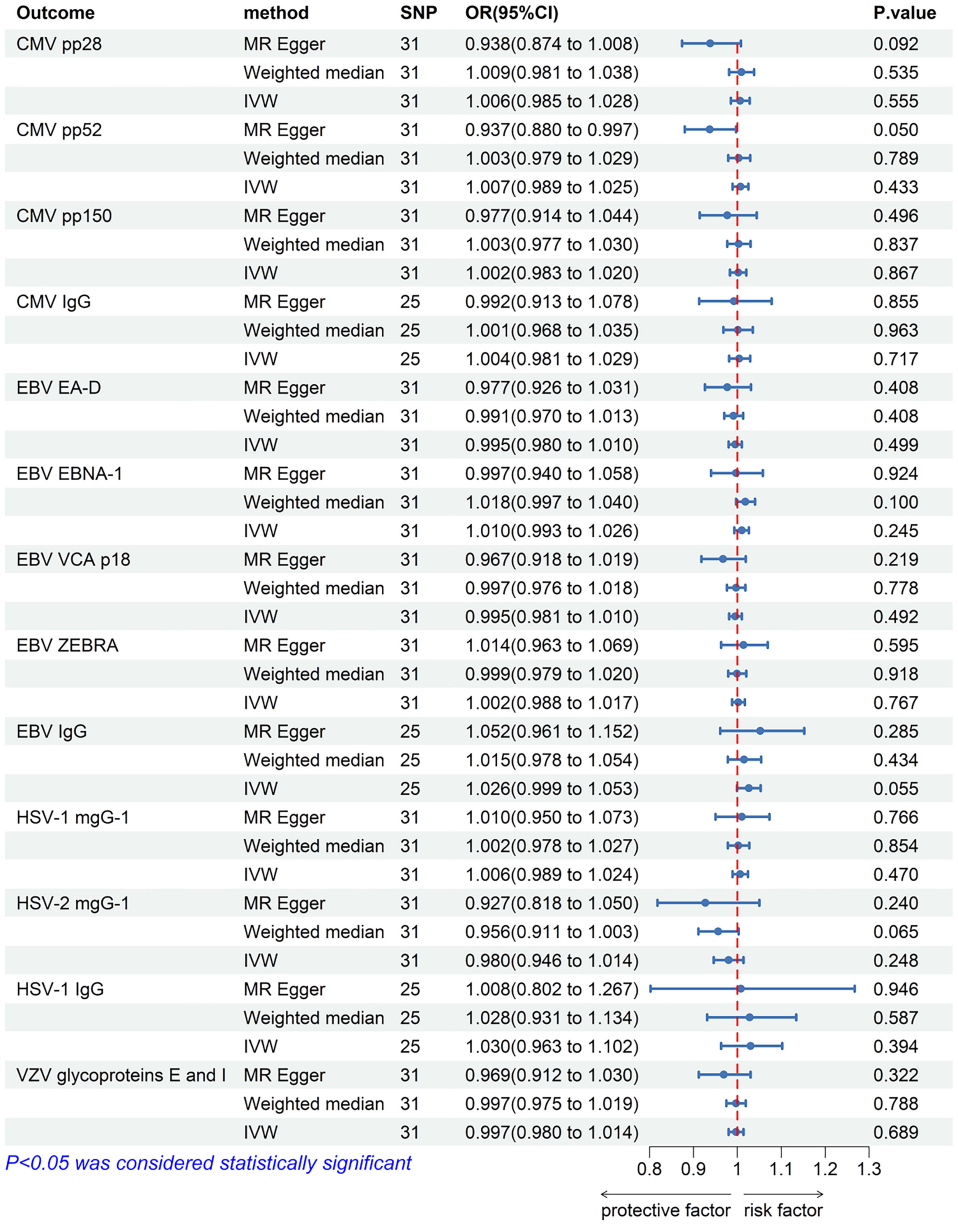
The forest plot of the causal relationship between allograft dysfunction and herpes virus infections. CMV, cytomegalovirus; EBV, Epstein-Barr virus; HSV, herpes simplex virus; VZV, varicella zoster virus; nSNP, number of single nucleotide polymorphisms; OR, odds ratio; CI, confidence interval; IVW, inverse variance weighted.

**Figure 9:**
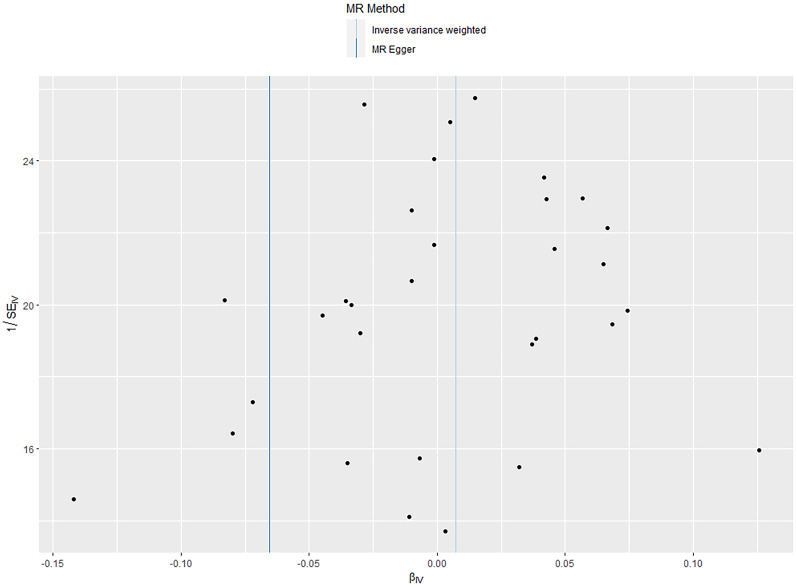
The funnel plot of allograft dysfunction and CMV pp52.

The IVW analysis results showed that there was no significant causal relationship between allograft dysfunction and the infection with any of the four tested herpes viruses (**Figure 8**). Similarly, neither the MR-Egger method nor the Weighted median method supported the conclusion that allograft dysfunction had a causal relationship with CMV, EBV, HSV or VZV. Although the MR-Egger analysis showed that allograft dysfunction may have an impact on the CMV pp52 antibody levels (OR = 0.937, 95% CI: 0.880-0.997, *p* = 0.050), the MR-Egger funnel plot (**Figure 9**) is not symmetrical. This indicates that this result is not robust, and therefore, the conclusion of a causal relationship between allograft dysfunction and CMVpp52 antibody levels is not supported.

## 4 Discussion

To our knowledge, this study is the first to assess the causal relationship between CMV, EBV, HSV and VZV and allograft dysfunction, and vice versa. Our findings support that there is a significant causal association between EBVEA-D antibody levels and allograft dysfunction, as well as an association between shingles and allograft dysfunction. Patients with higher levels of EBV EA-D antibodies or shingles are more likely to be at high risk for allograft dysfunction. These findings are robust based on the sensitivity analyses, which demonstrated that the methodology used in this project is less susceptible to confounding and reverse causality bias than many previous traditional observational studies (51).

EBNA-1, ZEBRA, EA-D and VCA-p18 are the four EBV proteins targeted in serology assays. Different serological characteristics may be related to the incubation and clearance periods of EBV infection (52, 53). For instance, IgM and IgG anti-EBV-CA (capsid antigen-CA) and anti-EA antibodies are produced during primary infection. In contrast, anti-EBNA-1 antibodies are detected during recovery and in advanced stages of primary EBV infection (54, 55). Our study found a significant association between anti-EA-D antibody levels and allograft dysfunction, suggesting that initial infection with EBV may increase the risk of allograft dysfunction. This increased risk may be associated with post-transplant lymphoproliferative disorders (PTLD). A statistical study showed that 63.6% of organ transplant recipients with EBV viremia were likely to progress to PTLD (56). In kidney transplantation, one study illustrates the association between subclinical cytomegalovirus and/or EBV viremia and decreased kidney function in patients under 5 years of age (57). Whether it is the direct viral cytopathic effect, indirect inflammatory effect, or the combination of various mechanisms that lead to allograft injury is still a key question that needs further investigation.

Both shingles and chickenpox are caused by VZV (58). However, chickenpox is caused by a primary VZV infection, whereas shingles is caused by the reactivation of latent VZV within the dorsal root ganglion (14). Therefore, the effects of the two infectious diseases on allograft dysfunction may differ. Primary chickenpox is an uncommon complication post-solid-organ transplant (SOT), except among pediatric transplant patients and those seronegative for VZV (59). As the majority of SOT recipients are seropositive for VZV, shingles occurs frequently following SOT, particularly among older recipients (≥65 years of age) and those receiving more intensive immunosuppression (59). Previous studies have also shown a high incidence of shingles among organ transplant recipients (60–62). A retrospective analysis showed that the incidence of shingles infection varied among different types of organ transplants: 17.1% in the heart, 14.0% in the lungs, 5.8% in the liver, and 9.2% in kidney transplant recipients (63). Our study further supports previous work and provides evidence that shingles is a risk factor for allograft dysfunction. Considering these results, we believe it is important monitor the zoster infection of organ transplant recipients promptly and take effective measures to prevent it.

Studies have shown that CMV is associated with increased mortality in patients following SOT (64). Helanterä et al. showed that CMV infection significantly reduced renal graft survival and renal function (65). Our study did not detect any causal relationship between CMV and allograft dysfunction, possibly due to insufficient data. Hence, further studies are needed to explore the relationship between CMV virus and allograft dysfunction.

Previous studies have shown that to prevent rejection after allogeneic organ transplantation, long-term immunosuppressive therapy is usually given to SOT recipients. This therapy often results in immune cell damage and lowered immunity in SOT recipients, making them more susceptible to herpes virus reactivation (66, 67). Therefore, the use of immunosuppressants or immune system conditions in SOT recipients is more likely than graft rejection or dysfunction to be associated causally with herpes virus infections. Further research is needed to confirm this conclusion.

There are some limitations to our study. First, the GWAS data used for the study may not have been comprehensive enough. The GWAS data we utilized came from populations of European descent, and as such, the applicability of our findings to other populations and regions remains to be determined. And the 23andMe database relies on self-reported questionnaires, so the dataset can only study symptomatic herpes virus infections. The datasets on antibody levels used in this study can provide a reference for asymptomatic virus herpes infections. Additionally, it was not possible to obtain data on all traits of herpes virus for MR analysis, such as anti-VZV IGg levels. Second, significant results were obtained only in IVW and MR-Egger. Therefore, further studies are needed to confirm and extend these findings, especially in larger clinical cohorts. Third, the lack of additional details regarding the failure and rejection of transplanted organs and tissues, such as transplant type, family medical history, genetic factors, age, sex, health awareness, other diseases, dietary habits, and the type and time of the rejection event, prevents us from conducting further stratified analysis. Hence, future studies should focus on collecting data from independent populations, obtain more SNPs, or expanding the sample size. Nevertheless, our work is the first to investigate the causal relationship between four herpes viruses and allograft dysfunction after tissue and organ transplantation using MR analyses, thus providing valuable insights into the field.

## 5 Conclusion

Overall, our study is the first to confirm, through Mendelian randomization, that initial infection with EBV or shingles in SOT recipients increases the risk of allograft dysfunction after organ and tissue transplantation. In addition, these results suggest that EBV and VZV play a crucial role in the pathological processes affecting allograft failure and rejection. This study provides valuable insights into the prevention and treatment of allograft dysfunction after organ and tissue transplantation.

## Data Availability

The original contributions presented in the study are included in the article/**Supplementary Material**, further inquiries can be directed to the corresponding author.

## References

[1] Black CK Termanini KM Aguirre O Hawksworth JS Sosin M. Solid organ transplantation in the 21(st) century. Ann Transl Med. (2018) 6:409. doi: 10.21037/atm 30498736 PMC6230860

[2] Martin-Gandul C Mueller NJ Pascual M Manuel O. The impact of infection on chronic allograft dysfunction and allograft survival after solid organ transplantation. Am J Transplant. (2015) 15:3024–40. doi: 10.1111/ajt.13486 26474168

[3] Roizmann B Desrosiers RC Fleckenstein B Lopez C Minson AC Studdert MJ. The family Herpesviridae: an update. The Herpesvirus Study Group of the International Committee on Taxonomy of Viruses. Arch Virol. (1992) 123:425–49. doi: 10.1007/BF01317276 1562239

[4] Duckworth A Longhurst HJ Paxton JK Scotton CJ. The role of herpes viruses in pulmonary fibrosis. Front Med. (2021) 8. doi: 10.3389/fmed.2021.704222 PMC8339799 34368196

[5] Fishman JA. Infection in organ transplantation. Am J Transplant. (2017) 17:856–79. doi: 10.1111/ajt.14208 28117944

[6] Götzinger P Sautner T Wamser P Gebhard B Barlan M Steininger R. [Early postoperative infections after liver transplantation–pathogen spectrum and risk factors]. Wien Klin Wochenschr. (1996) 108:795–801. 9092210

[7] Jenkins FJ Rowe DT Rinaldo CR Jr. Herpesvirus infections in organ transplant recipients. Clin Diagn Lab Immunol. (2003) 10:1–7. doi: 10.1128/CDLI.10.1.1-7.2003 12522031 PMC145294

[8] Lumbreras C Fernandez I Velosa J Munn S Sterioff S Paya CV. Infectious complications following pancreatic transplantation: incidence, microbiological and clinical characteristics, and outcome. Clin Infect Dis. (1995) 20:514–20. doi: 10.1093/clinids/20.3.514 7756469

[9] Malahe SRK van Kampen JJA Manintveld OC Hoek RAS den Hoed CM Baan CC. Current perspectives on the management of herpesvirus infections in solid organ transplant recipients. Viruses. (2023) 15:1595. doi: 10.3390/v15071595 37515280 PMC10383436

[10] Razonable RR Humar A. Cytomegalovirus in solid organ transplant recipients-Guidelines of the American Society of Transplantation Infectious Diseases Community of Practice. Clin Transplant. (2019) 33:e13512. doi: 10.1111/ctr.13512 30817026

[11] Allen UD Preiksaitis JK. Epstein-barr virus and posttransplant lymphoproliferative disorder in solid organ transplantation. Am J Transplant. (2013) 13:107–20. doi: 10.1111/ajt.12104 23465004

[12] San-Juan R Manuel O Hirsch HH Fernández-Ruiz M López-Medrano F Comoli P. Current preventive strategies and management of Epstein–Barr virus-related post-transplant lymphoproliferative disease in solid organ transplantation in Europe. Results of the ESGICH Questionnaire-based Cross-sectional Survey. Clin Microbiol Infection. (2015) 21:604.e1–.e9. doi: 10.1016/j.cmi.2015.02.002 25686696

[13] Green M. Management of epstein–barr virus-induced post-transplant lymphoproliferative disease in recipients of solid organ transplantation. Am J Transplant. (2001) 1:103–8. doi: 10.1034/j.1600-6143.2001.10202.x 12099356

[14] Zuckerman RA Limaye AP. Varicella zoster virus (VZV) and herpes simplex virus (HSV) in solid organ transplant patients. Am J Transplant. (2013) 13:55–66. doi: 10.1111/ajt.12003 23347214

[15] Son M Lee M Sung G Lee T Shin Y Cho H. Bioactive activities of natural products against herpesvirus infection. J Microbiol. (2013) 51:545–51. doi: 10.1007/s12275-013-3450-9 24173639

[16] Skoreński M Sieńczyk M. Anti-herpesvirus agents: a patent and literature review (2003 to present). Expert Opin Ther patents. (2014) 24:925–41. doi: 10.1517/13543776.2014.927442 25010889

[17] Li R Hayward S. Potential of protein kinase inhibitors for treating herpesvirus-associated disease. Trends Microbiol. (2013) 21:286–95. doi: 10.1016/j.tim.2013.03.005 PMC3674143 23608036

[18] Sekula P Del Greco MF Pattaro C Köttgen A. Mendelian randomization as an approach to assess causality using observational data. J Am Soc Nephrol. (2016) 27:3253–65. doi: 10.1681/ASN.2016010098 PMC5084898 27486138

[19] Davey Smith G Hemani G. Mendelian randomization: genetic anchors for causal inference in epidemiological studies. Hum Mol Genet. (2014) 23:R89–98. doi: 10.1093/hmg/ddu328 PMC4170722 25064373

[20] Bowden J Holmes MV. Meta-analysis and Mendelian randomization: A review. Res Synthesis Methods. (2019) 10:486–96. doi: 10.1002/jrsm.1346 PMC6973275 30861319

[21] Lawlor DA Harbord RM Sterne JA Timpson N Davey Smith G. Mendelian randomization: using genes as instruments for making causal inferences in epidemiology. Stat Med. (2008) 27:1133–63. doi: 10.1002/sim.3034 17886233

[22] Walker VM Zheng J Gaunt TR Smith GD. Phenotypic causal inference using genome-wide association study data: mendelian randomization and beyond. Annu Rev BioMed Data Sci. (2022) 5:1–17. doi: 10.1146/annurev-biodatasci-122120-024910 35363507 PMC7614231

[23] Davey Smith G Ebrahim S. ‘Mendelian randomization’: can genetic epidemiology contribute to understanding environmental determinants of disease?*. Int J Epidemiol. (2003) 32:1–22. doi: 10.1093/ije/dyg070 12689998

[24] Agostini S Yan H Zhu C Jin X Feng G. Mendelian randomization reveals no correlations between herpesvirus infection and idiopathic pulmonary fibrosis. PloS One. (2023) 18:e0295082. doi: 10.1371/journal.pone.0295082 38015883 PMC10683991

[25] Zhang M Ming Y Du Y Xin Z. Two-sample Mendelian randomization study does not reveal a significant relationship between cytomegalovirus (CMV) infection and autism spectrum disorder. BMC Psychiatry. (2023) 23:559. doi: 10.1186/s12888-023-05035-w 37533011 PMC10394766

[26] Huang S-Y Yang Y-X Kuo K Li H-Q Shen X-N Chen S-D. Herpesvirus infections and Alzheimer’s disease: a Mendelian randomization study. Alzheimer's Res Ther. (2021) 13:1–8. doi: 10.1186/s13195-021-00905-5 PMC8464096 34560893

[27] Zhao Y Du D Wei L Chen Z. Value of blood lipid in predicting graft dysfunction after organ and tissue transplantation: A study of Mendelian randomization. Heliyon. (2023) 9:e20230. doi: 10.1016/j.heliyon.2023.e20230 37809918 PMC10559986

[28] Tian C Hromatka BS Kiefer AK Eriksson N Noble SM Tung JY. Genome-wide association and HLA region fine-mapping studies identify susceptibility loci for multiple common infections. Nat Commun. (2017) 8:599. doi: 10.1038/s41467-017-00257-5 28928442 PMC5605711

[29] Elsworth B Lyon M Alexander T Liu Y Matthews P Hallett J. The MRC IEU OpenGWAS data infrastructure. bioRxiv. (2020). doi: 10.1101/2020.08.10.244293

[30] IEU Open GWAS project . Available online at: https://gwas.mrcieu.ac.uk/.

[31] Butler-Laporte G Kreuzer D Nakanishi T Harroud A Forgetta V Richards JB. Genetic determinants of antibody-mediated immune responses to infectious diseases agents: A genome-wide and HLA association study. Open Forum Infect Dis. (2020) 7:ofaa450. doi: 10.1093/ofid/ofaa450 33204752 PMC7641500

[32] FinnGen . Available online at: https://www.finngen.fi/.

[33] Huang Y Li X Ye W. Application of mendelian randomization to study the causal relationship between smoking and the risk of chronic obstructive pulmonary disease. PloS One. (2023) 18:e0288783. doi: 10.1371/journal.pone.0288783 37506114 PMC10381044

[34] Chen Y Shen J Wu Y Ni M Deng Y Sun X. Tea consumption and risk of lower respiratory tract infections: a two-sample mendelian randomization study. Eur J Nutr. (2023) 62:385–93. doi: 10.1007/s00394-022-02994-w PMC9427168 36042048

[35] Pierce BL Ahsan H Vanderweele TJ. Power and instrument strength requirements for Mendelian randomization studies using multiple genetic variants. Int J Epidemiol. (2011) 40:740–52. doi: 10.1093/ije/dyq151 PMC3147064 20813862

[36] Jiang X Zhou R He Y Zhu T Zhang W. Causal effect of serum 25-hydroxyvitamin D levels on low back pain: A two-sample mendelian randomization study. Front Genet. (2022) 13:1001265. doi: 10.3389/fgene.2022.1001265 36212121 PMC9534573

[37] Andrews SJ Goate A Anstey KJ. Association between alcohol consumption and Alzheimer's disease: A Mendelian randomization study. Alzheimer's Dementia. (2020) 16:345–53. doi: 10.1016/j.jalz.2019.09.086 PMC7057166 31786126

[38] Kamat MA Blackshaw JA Young R Surendran P Burgess S Danesh J. PhenoScanner V2: an expanded tool for searching human genotype-phenotype associations. Bioinformatics. (2019) 35:4851–3. doi: 10.1093/bioinformatics/btz469 PMC6853652 31233103

[39] Young Robin Blackshaw James Staley James. PhenoScanner: A database of human genotype-phenotype associations. Genet Epidemiol. (2016) 32:3207–9. doi: 10.1093/bioinformatics/btw373 PMC5048068 27318201

[40] PhenoScanner. Available online at: http://www.phenoscanner.medschl.cam.ac.uk/.

[41] Hemani G Zheng J Elsworth B Wade KH Haberland V Baird D. The MR-Base platform supports systematic causal inference across the human phenome. Elife. (2018) 7:e34408. doi: 10.7554/eLife.34408 29846171 PMC5976434

[42] R Core Team. A Language and Environment for Statistical Computing. Venna: R Foundation for Statistical Computing (2022).

[43] Burgess S Scott RA Timpson NJ Davey Smith G Thompson SG. Using published data in Mendelian randomization: a blueprint for efficient identification of causal risk factors. Eur J Epidemiol. (2015) 30:543–52. doi: 10.1007/s10654-015-0011-z PMC4516908 25773750

[44] Luo S Li W Li Q Zhang M Wang X Wu S. Causal effects of gut microbiota on the risk of periodontitis: a two-sample Mendelian randomization study. Front Cell Infect Microbiol. (2023) 13:1160993. doi: 10.3389/fcimb.2023.1160993 37305424 PMC10248501

[45] Bowden J Davey Smith G Haycock PC Burgess S. Consistent estimation in mendelian randomization with some invalid instruments using a weighted median estimator. Genet Epidemiol. (2016) 40:304–14. doi: 10.1002/gepi.21965 PMC4849733 27061298

[46] Bowden J Davey Smith G Burgess S. Mendelian randomization with invalid instruments: effect estimation and bias detection through Egger regression. Int J Epidemiol. (2015) 44:512–25. doi: 10.1093/ije/dyv080 PMC4469799 26050253

[47] Fang P Liu X Qiu Y Wang Y Wang D Zhao J. Exploring causal correlations between inflammatory cytokines and ankylosing spondylitis: a bidirectional mendelian-randomization study. Front Immunol. (2023) 14. doi: 10.3389/fimmu.2023.1285106 PMC10694192 38054001

[48] Zheng H Shi Y Liang J Lu L Chen M. Modifiable factors for migraine prophylaxis: A mendelian randomization analysis. Front Pharmacol. (2023) 14:1010996. doi: 10.3389/fphar.2023.1010996 36713835 PMC9878312

[49] Li R Peng L Deng D Li G Wu S. Potential causal association between aspirin use and erectile dysfunction in European population: a Mendelian randomization study. Front Endocrinol (Lausanne). (2023) 14:1329847. doi: 10.3389/fendo.2023.1329847 38260164 PMC10800513

[50] Burgess S Thompson SG. Interpreting findings from Mendelian randomization using the MR-Egger method. Eur J Epidemiol. (2017) 32:377–89. doi: 10.1007/s10654-017-0255-x PMC5506233 28527048

[51] Davies NM Holmes MV Davey Smith G. Reading Mendelian randomization studies: a guide, glossary, and checklist for clinicians. Bmj. (2018) 362:k601. doi: 10.1136/bmj.k601 30002074 PMC6041728

[52] Erre GL Mameli G Cossu D Muzzeddu B Piras C Paccagnini D. Increased epstein-barr virus DNA load and antibodies against EBNA1 and EA in sardinian patients with rheumatoid arthritis. Viral Immunol. (2015) 28:385–90. doi: 10.1089/vim.2015.0035 26083265

[53] Westergaard MW Draborg AH Troelsen L Jacobsen S Houen G. Isotypes of Epstein-Barr virus antibodies in rheumatoid arthritis: association with rheumatoid factors and citrulline-dependent antibodies. BioMed Res Int. (2015) 2015:472174. doi: 10.1155/2015/472174 26000294 PMC4426970

[54] Miljanovic D Cirkovic A Jermic I Basaric M Lazarevic I Grk M. Markers of epstein-barr virus infection in association with the onset and poor control of rheumatoid arthritis: A prospective cohort study. Microorganisms. (2023) 11:1958. doi: 10.3390/microorganisms11081958 37630516 PMC10459700

[55] De Paschale M Clerici P. Serological diagnosis of Epstein-Barr virus infection: Problems and solutions. World J Virol. (2012) 1:31–43. doi: 10.5501/wjv.v1.i1.31 24175209 PMC3782265

[56] Pullerits K Garland S Rengarajan S Guiver M Chinnadurai R Middleton RJ. Kidney transplant-associated viral infection rates and outcomes in a single-center cohort. Viruses. (2022) 14:2406. doi: 10.3390/v14112406 36366504 PMC9695979

[57] Li L Chaudhuri A Weintraub LA Hsieh F Shah S Alexander S. Subclinical cytomegalovirus and Epstein–Barr virus viremia are associated with adverse outcomes in pediatric renal transplantation. Pediatr Transplant. (2007) 11:187–95. doi: 10.1111/j.1399-3046.2006.00641.x 17300499

[58] Zerboni L Sen N Oliver SL Arvin AM. Molecular mechanisms of varicella zoster virus pathogenesis. Nat Rev Microbiol. (2014) 12:197–210. doi: 10.1038/nrmicro3215 24509782 PMC4066823

[59] Pergam SA Limaye AP. Varicella zoster virus in solid organ transplantation: Guidelines from the American Society of Transplantation Infectious Diseases Community of Practice. Clin Transplant. (2019) 33:e13622. doi: 10.1111/ctr.13622 31162727

[60] Arness T Pedersen R Dierkhising R Kremers W Patel R. Varicella zoster virus-associated disease in adult kidney transplant recipients: incidence and risk-factor analysis. Transpl Infect Dis. (2008) 10:260–8. doi: 10.1111/j.1399-3062.2007.00289.x 18086277

[61] Kwon DE Lee HS Lee KH La Y Han SH Song YG. Incidence of herpes zoster in adult solid organ transplant recipients: A meta-analysis and comprehensive review. Transpl Infect Dis. (2021) 23:e13674. doi: 10.1111/tid.13674 34153168

[62] Vink P Ramon Torrell JM Sanchez Fructuoso A Kim SJ Kim SI Zaltzman J. Immunogenicity and safety of the adjuvanted recombinant zoster vaccine in chronically immunosuppressed adults following renal transplant: A phase 3, randomized clinical trial. Clin Infect Dis. (2020) 70:181–90. doi: 10.1093/ofid/ofx163.1044 PMC6938982 30843046

[63] Kho MML Roest S Bovée DM Metselaar HJ Hoek RAS van der Eijk AA. Herpes zoster in solid organ transplantation: incidence and risk factors. Front Immunol. (2021) 12:645718. doi: 10.3389/fimmu.2021.645718 33815403 PMC8012754

[64] Bosch W Heckman MG Diehl NN Shalev JA Pungpapong S Hellinger WC. Association of cytomegalovirus infection and disease with death and graft loss after liver transplant in high-risk recipients. Am J Transplant. (2011) 11:2181–9. doi: 10.1111/j.1600-6143.2011.03618.x 21827609

[65] Helanterä I Koskinen P Finne P Loginov R Kyllönen L Salmela K. Persistent cytomegalovirus infection in kidney allografts is associated with inferior graft function and survival. Transpl Int. (2006) 19:893–900. doi: 10.1111/j.1432-2277.2006.00364.x 17018124

[66] Schröder C Enders D Schink T Riedel O. Incidence of herpes zoster amongst adults varies by severity of immunosuppression. J Infect. (2017) 75:207–15. doi: 10.1016/j.jinf.2017.06.010 28676411

[67] Muñoz-Quiles C López-Lacort M Díez-Domingo J Orrico-Sánchez A. Herpes zoster risk and burden of disease in immunocompromised populations: a population-based study using health system integrated databases, 2009-2014. BMC Infect Dis. (2020) 20:905. doi: 10.1186/s12879-020-05648-6 33256624 PMC7708196

