# Supplementary material for: Causality between herpes virus infections and allograft dysfunction after tissue and organ transplantation: a two-sample bidirectional Mendelian randomization study
Source: Front Immunol. 2024 Aug 14;15:1411771. doi: 10.3389/fimmu.2024.1411771 (PMC11359570; doi:10.3389/fimmu.2024.1411771)
Supplement: Supplementary file 1 [file DataSheet1.pdf]

Table S1. Brief description and access link of datasets utilized in the Mendelian randomization study.

Phenotype	GWAS ID	Source	Sample size (Cases/Controls)	Population	Access Link
Mononucleosis	mononucleosis	23andMe cohort	17457/68446	European	https://doi.org/10.1038/s41467-017-00257-5
Cold scores	cold scores		25108/63332	European	
Chickenpox	chickenpox		107769/15982	European	
Shingles	shingles		16711/118152	European	
CMV pp28 antibody levels	ebi-a-GCST90006894	UK Biobank cohort	5,087	European	https://doi.org/10.1093/ofid/ofaa450
CMV pp52 antibody levels	ebi-a-GCST90006895		5,681	European	
CMV pp150 antibody levels	ebi-a-GCST90006896		5,136	European	
EBV EA-D antibody levels	ebi-a-GCST90006898		7,763	European	
EBV EBNA-1 antibody levels	ebi-a-GCST90006899		7,972	European	
EBV VCA p18 antibody levels	ebi-a-GCST90006900		8,518	European	
EBV ZEBRA antibody levels	ebi-a-GCST90006901		8,191	European	
HSV-1 mgG-1 antibody levels	ebi-a-GCST90006918		6,199	European	
HSV-2 mgG-1 antibody levels	ebi-a-GCST90006920		1,382	European	
VZV glycoproteins E and I antibody levels	ebi-a-GCST90006929		7,595	European	
Anti-CMV IgG levels	ieu-b-4900	IEU OPEN GWAS	5,010	European	https://gwas.mrcieu.ac.uk/datasets/ieu-b-4900/
Anti-EBV IgG levels	ieu-b-4901		5,010	European	https://gwas.mrcieu.ac.uk/datasets/ieu-b-4901/
Anti-HSV-1 IgG levels	ieu-b-4906		683	European	https://gwas.mrcieu.ac.uk/datasets/ieu-b-4906/
Failure and rejection of transplanted organs and tissues	FAILU_REJEC_TRANSPLANTED_ORGANS_TISSU	FinnGen cohort	209/278724	European	https://r7.finngen.fi/pheno/ST19_FAILU_REJEC_TRANSPLANTED_ORGANS_TISSU

Abbreviations: CMV, cytomegalovirus; EBV, Epstein-Barr virus; HSV, herpes simplex; VZV, Varicella zoster virus; EA, EBV early antigen; EBNA-1, EBV nuclear antigen-1; VCA, EBV viral capsid antigen; IgG, immunoglobulin G.

Table S2. A list of confounders removed by phenoscanner in the forward MR.

GWAS ID	confounder	effect_allele	other_allele
chickenpox	rs4246519	G	A
ebi-a-GCST90006894	rs558240	A	G
ieu-b-4900	rs7761068	G	A

Table S3. Results of MR Pleiotropy and heterogeneity tests.

Exposure	Outcome	MR-PRESSO		Pleiotropy test	Heterogeneity test	
		Distortion test	Global test	Egger intercept	Cochran's Q test	Rucker's Q test
					P-value	P-value
		Outliers	P-value	P-value	IVW	MR-Egger
mononucleosis	FAILU_REJEC_TP_ORGANS_TISSU	NA	0.786	0.791	0.762	0.736
cold scores		NA	0.400	0.167	0.404	0.439
chickenpox		NA	0.438	0.539	0.464	0.441
shingles		NA	0.807	0.052	0.792	0.880
ebi-a-GCST90006894		NA	0.002	0.450	0.002	0.002
ebi-a-GCST90006895		NA	0.115	0.443	0.113	0.108
ebi-a-GCST90006896		NA	0.746	0.392	0.808	0.804
ebi-a-GCST90006898		NA	0.527	0.046	0.533	0.644
ebi-a-GCST90006899		NA	0.264	0.074	0.291	0.359
ebi-a-GCST90006900		NA	0.071	0.759	0.076	0.067
ebi-a-GCST90006901		NA	0.402	0.894	0.410	0.381
ebi-a-GCST90006918		NA	0.177	0.551	0.211	0.198
ebi-a-GCST90006920		NA	0.735	0.120	0.751	0.791
ebi-a-GCST90006929		NA	0.165	0.841	0.176	0.157
ieu-b-4900		NA	0.913	0.356	0.905	0.905
ieu-b-4901		NA	0.454	0.692	0.468	0.440
ieu-b-4906		NA	0.040	0.100	0.0457	0.160
FAILU_REJEC_TP_ORGANS_TISSU	ebi-a-GCST90006894	NA	0.168	0.056	0.152	0.265
	ebi-a-GCST90006895	NA	0.457	0.025	0.425	0.667
	ebi-a-GCST90006896	NA	0.776	0.449	0.771	0.756
	ebi-a-GCST90006898	NA	0.515	0.503	0.502	0.474
	ebi-a-GCST90006899	NA	0.263	0.668	0.242	0.212
	ebi-a-GCST90006900	NA	0.516	0.277	0.497	0.509
	ebi-a-GCST90006901	NA	0.619	0.640	0.609	0.569
	ebi-a-GCST90006918	NA	0.935	0.919	0.915	0.891
	ebi-a-GCST90006920	NA	0.714	0.369	0.690	0.684
	ebi-a-GCST90006929	NA	0.177	0.358	0.170	0.169
	ieu-b-4900	NA	0.507	0.764	0.488	0.435
	ieu-b-4901	NA	0.275	0.577	0.265	0.235
	ieu-b-4906	NA	0.733	0.100	0.720	0.669

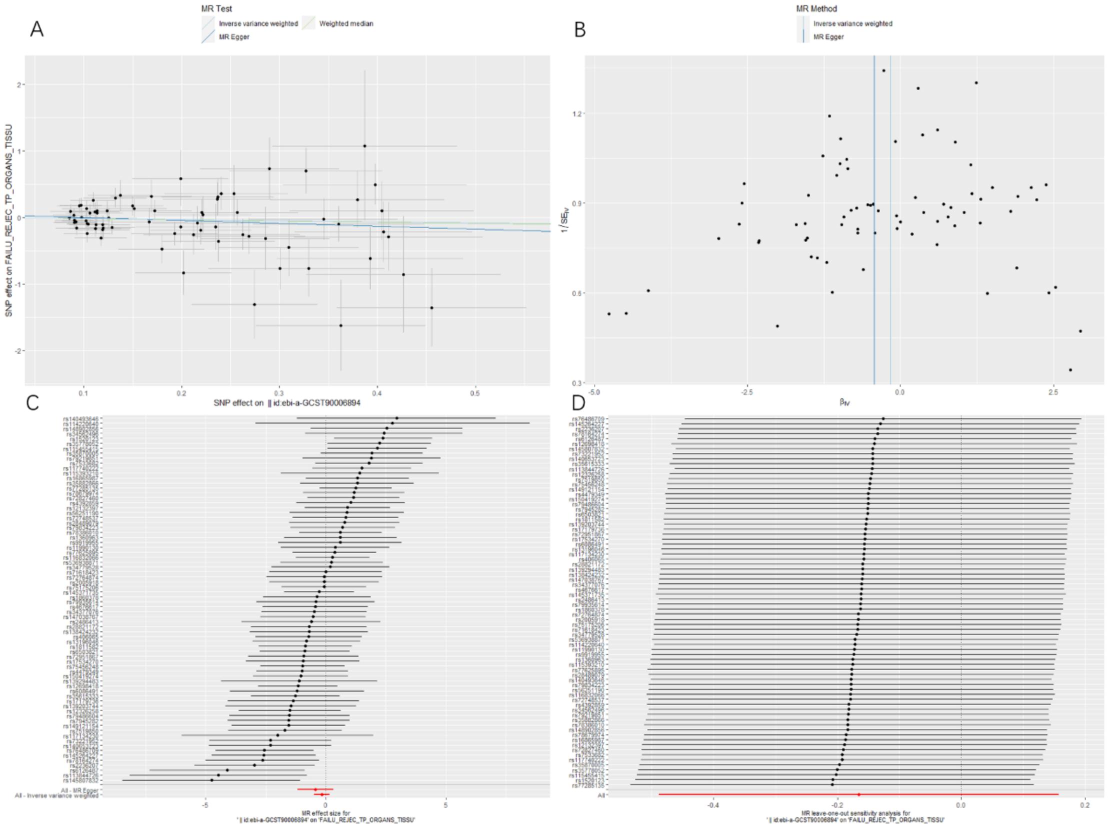

Figure S1. The scatter plot (A), funnel plot (B), forest plot (C) and leave-one-out plot (D) for the association of CMV pp28 antibody levels on allograft dysfunction in the forward MR analysis.

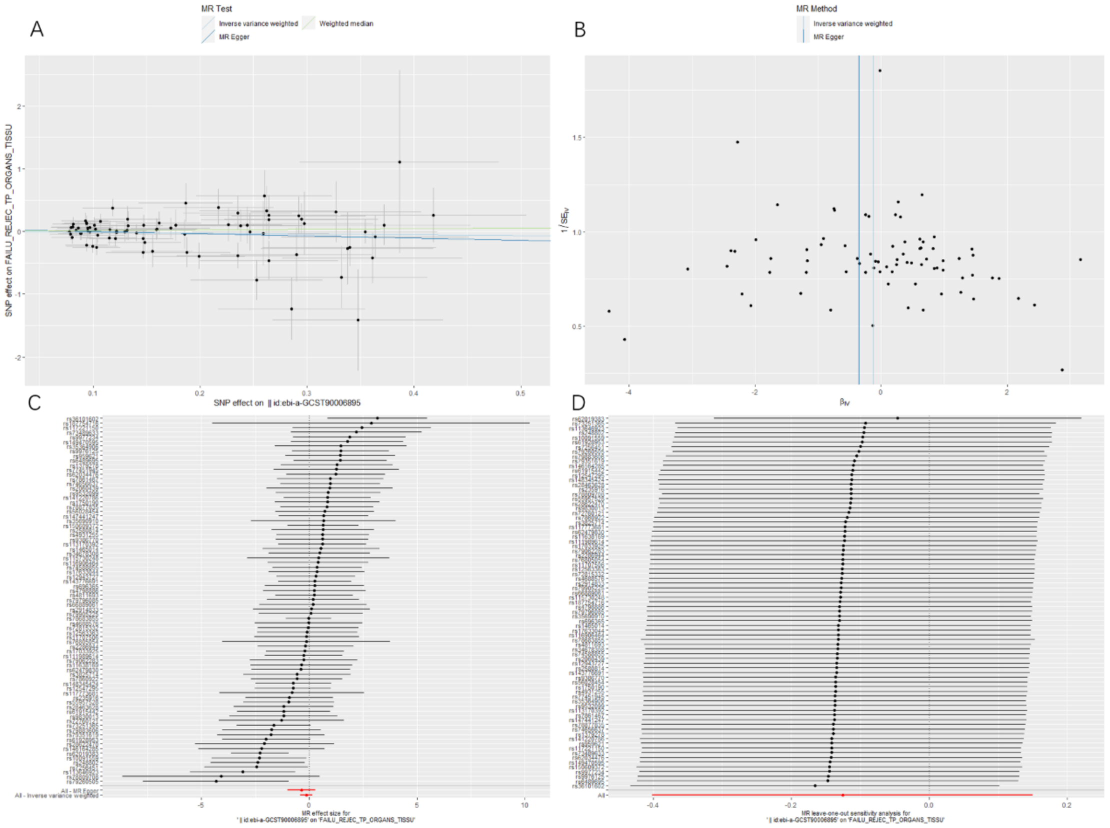

Figure S2. The scatter plot (A), funnel plot (B), forest plot (C) and leave-one-out plot (D) for the association of CMV pp52 antibody levels on allograft dysfunction in the forward MR analysis.

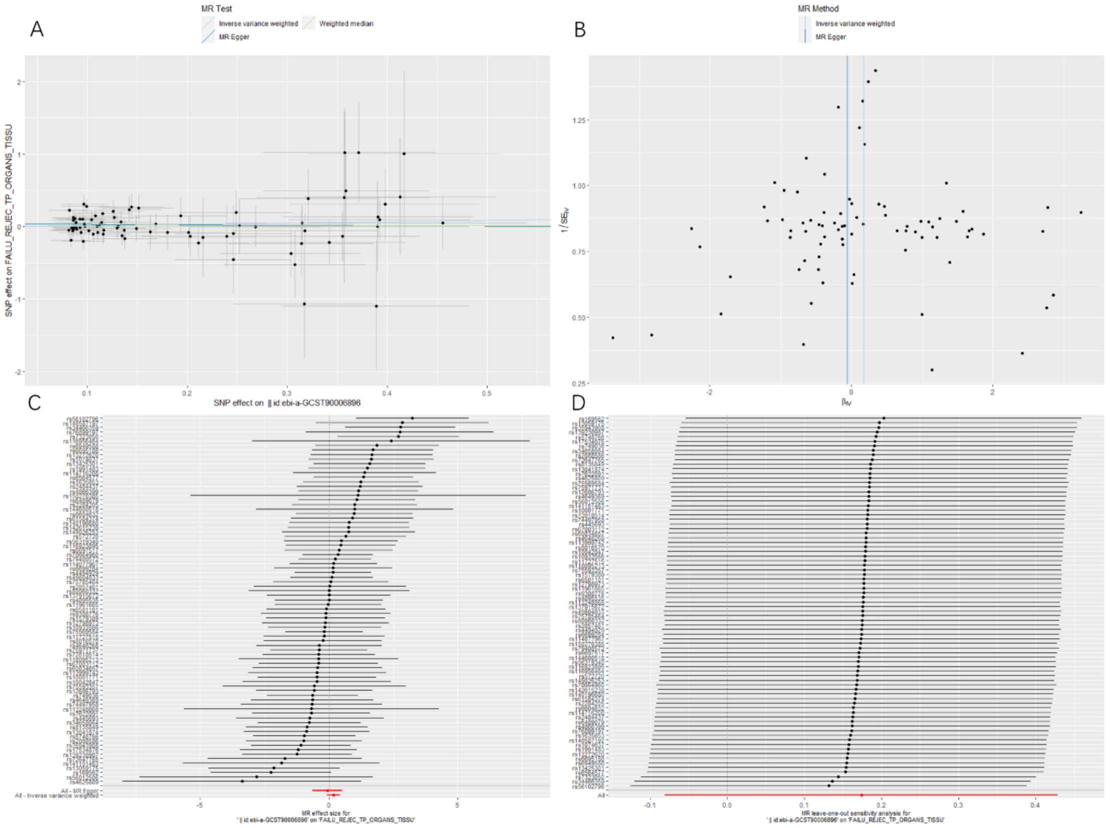

Figure S3. The scatter plot (A), funnel plot (B), forest plot (C) and leave-one-out plot (D) for the association of CMV pp150 antibody levels on allograft dysfunction in the forward MR analysis.

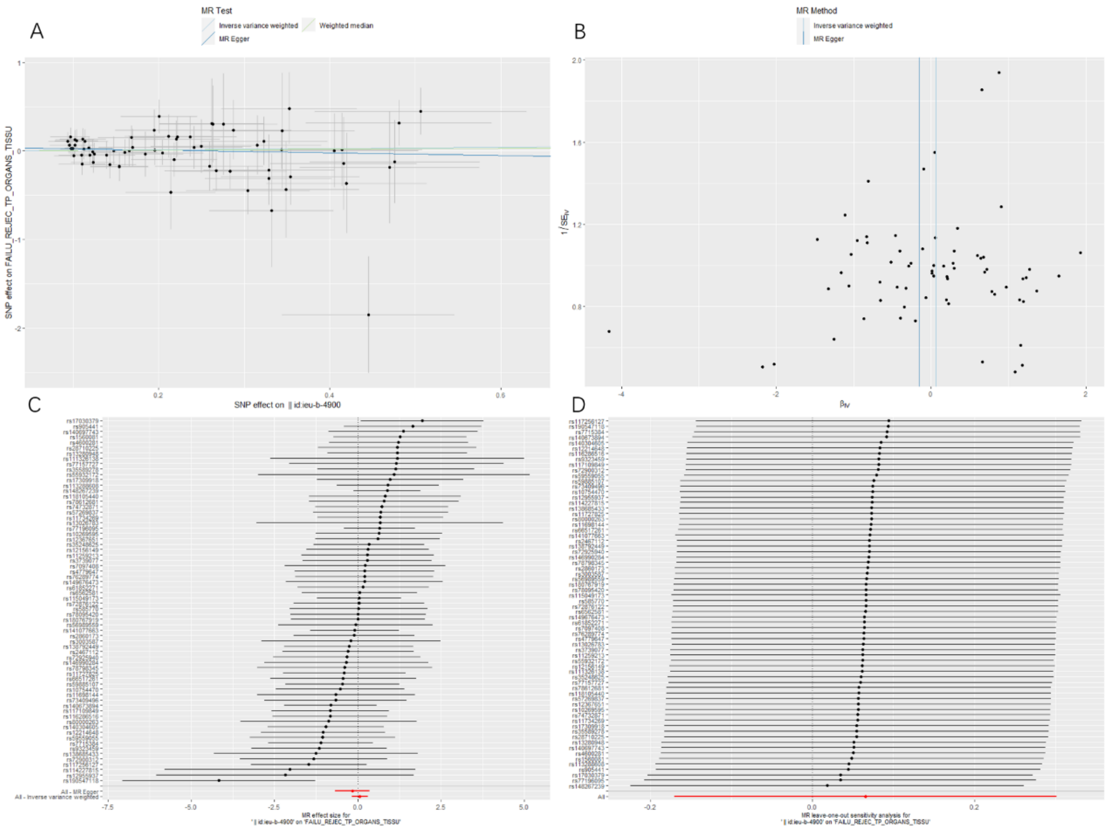

Figure S4. The scatter plot (A), funnel plot (B), forest plot (C) and leave-one-out plot (D) for the association of anti-CMV IgG levels on allograft dysfunction in the forward MR analysis.

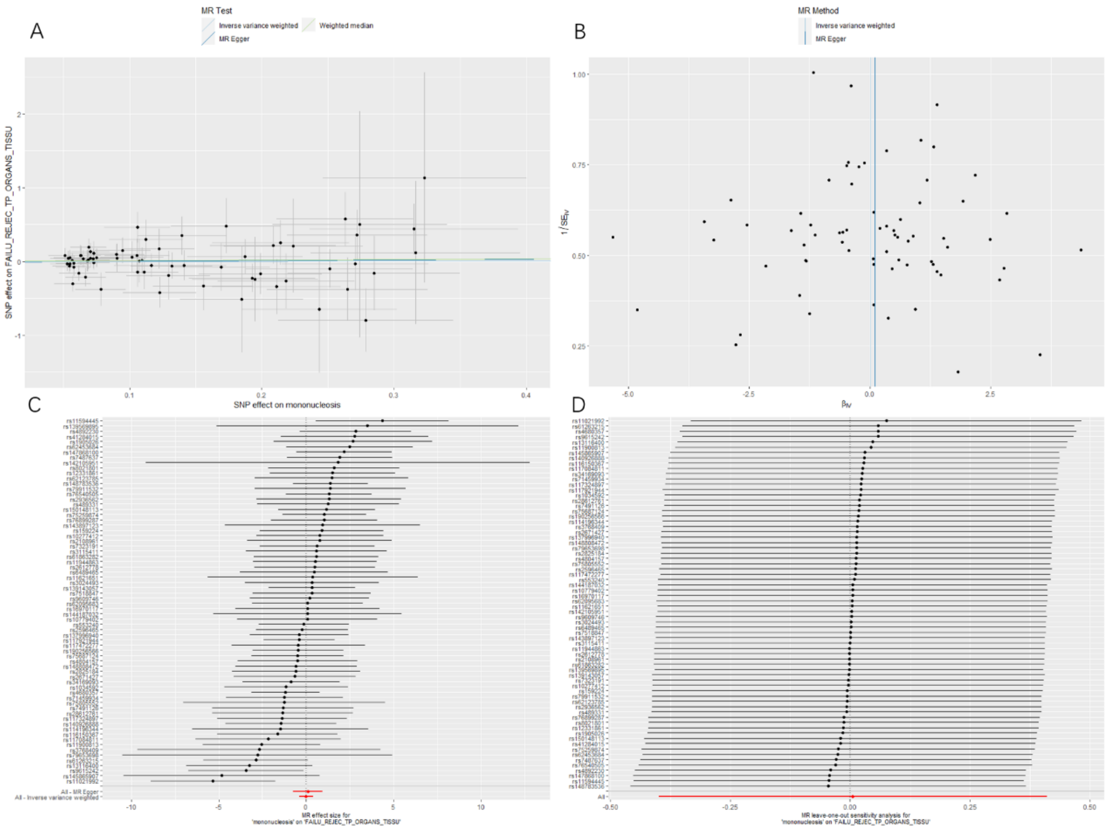

Figure S5. The scatter plot (A), funnel plot (B), forest plot (C) and leave-one-out plot (D) for the association of mononucleosis on allograft dysfunction in the forward MR analysis.

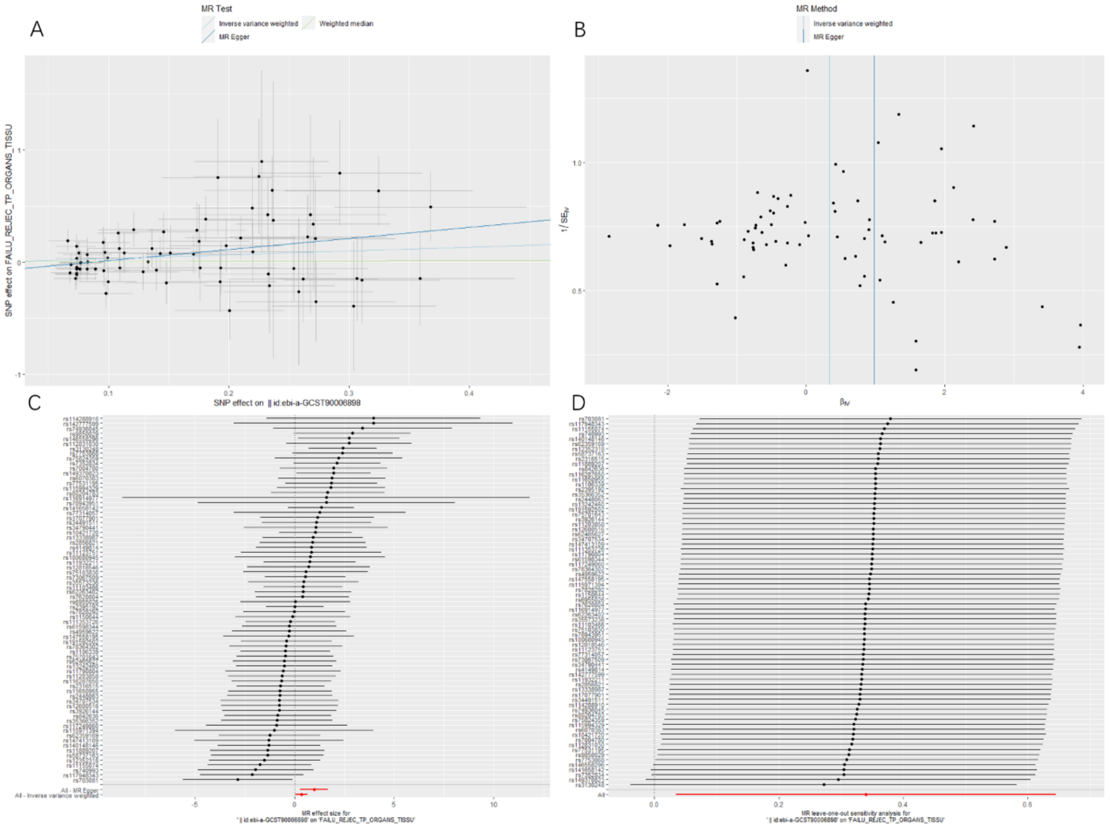

Figure S6. The scatter plot (A), funnel plot (B), forest plot (C) and leave-one-out plot (D) for the association of EBV EA-D antibody levels on allograft dysfunction in the forward MR analysis.

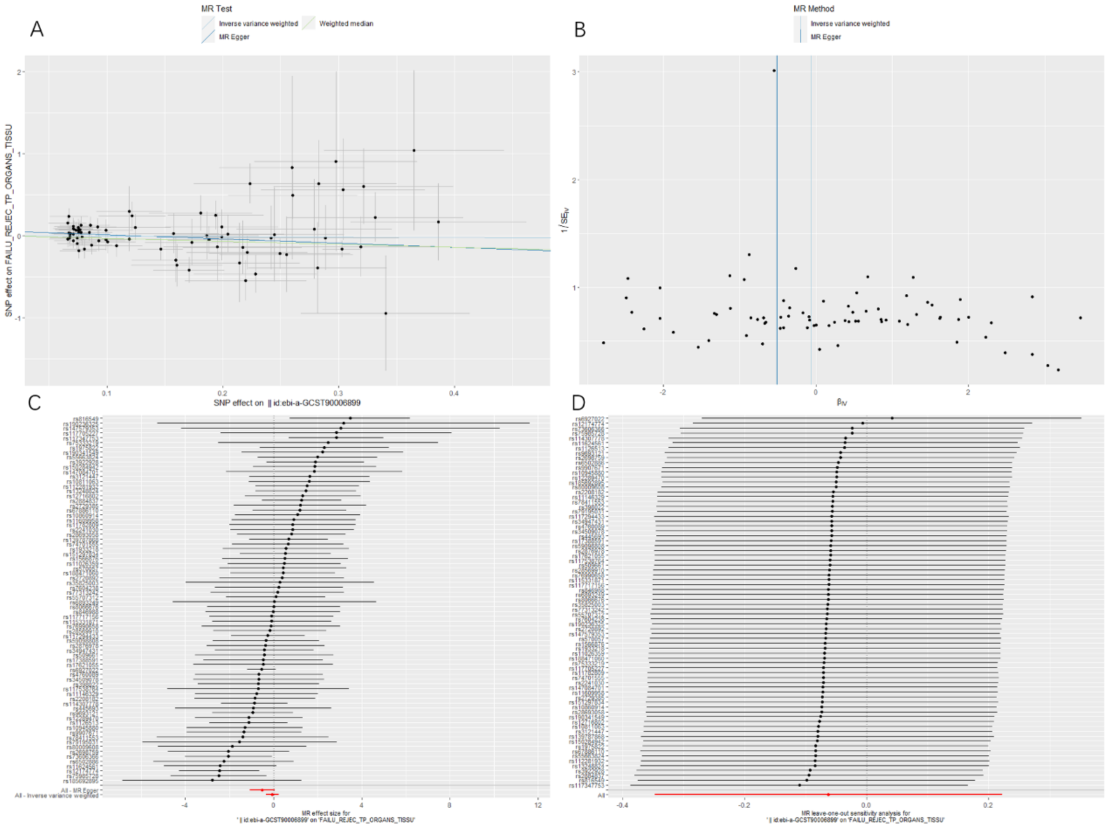

Figure S7. The scatter plot (A), funnel plot (B), forest plot (C) and leave-one-out plot (D) for the association of EBV EBNA-1 antibody levels on allograft dysfunction in the forward MR analysis.

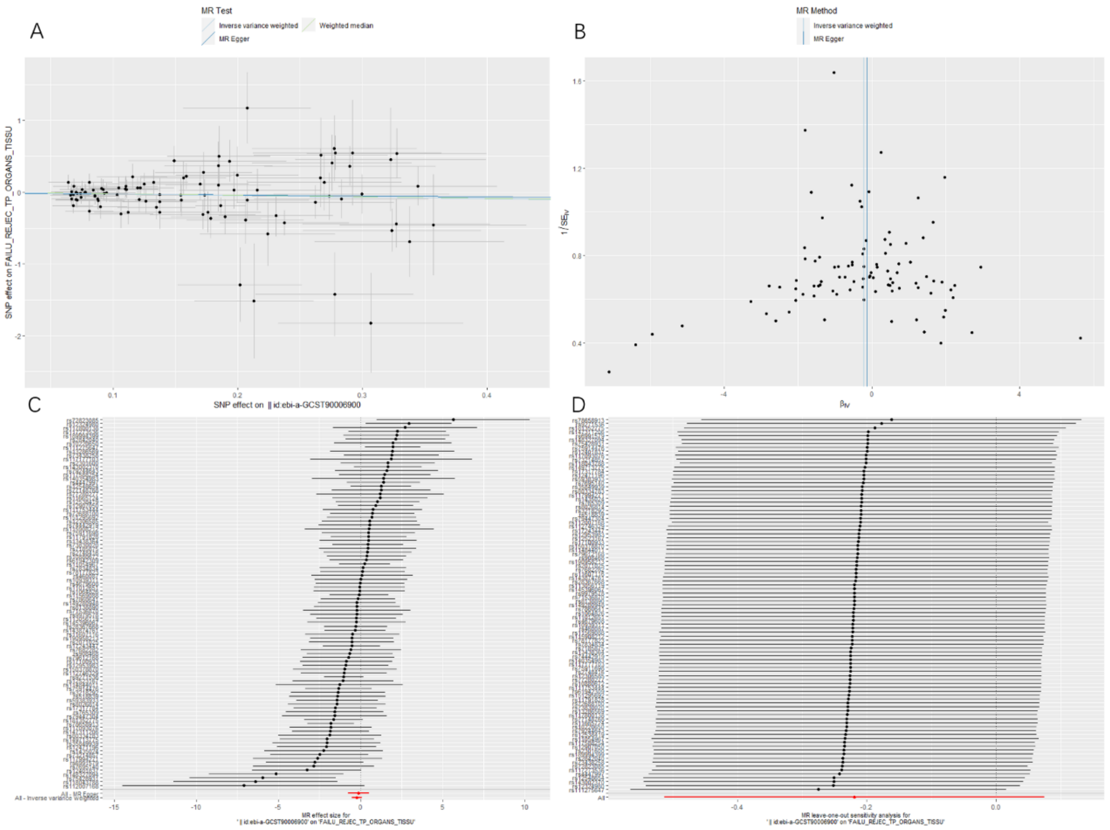

Figure S8. The scatter plot (A), funnel plot (B), forest plot (C) and leave-one-out plot (D) for the association of EBV VCA p18 antibody levels on allograft dysfunction in the forward MR analysis.

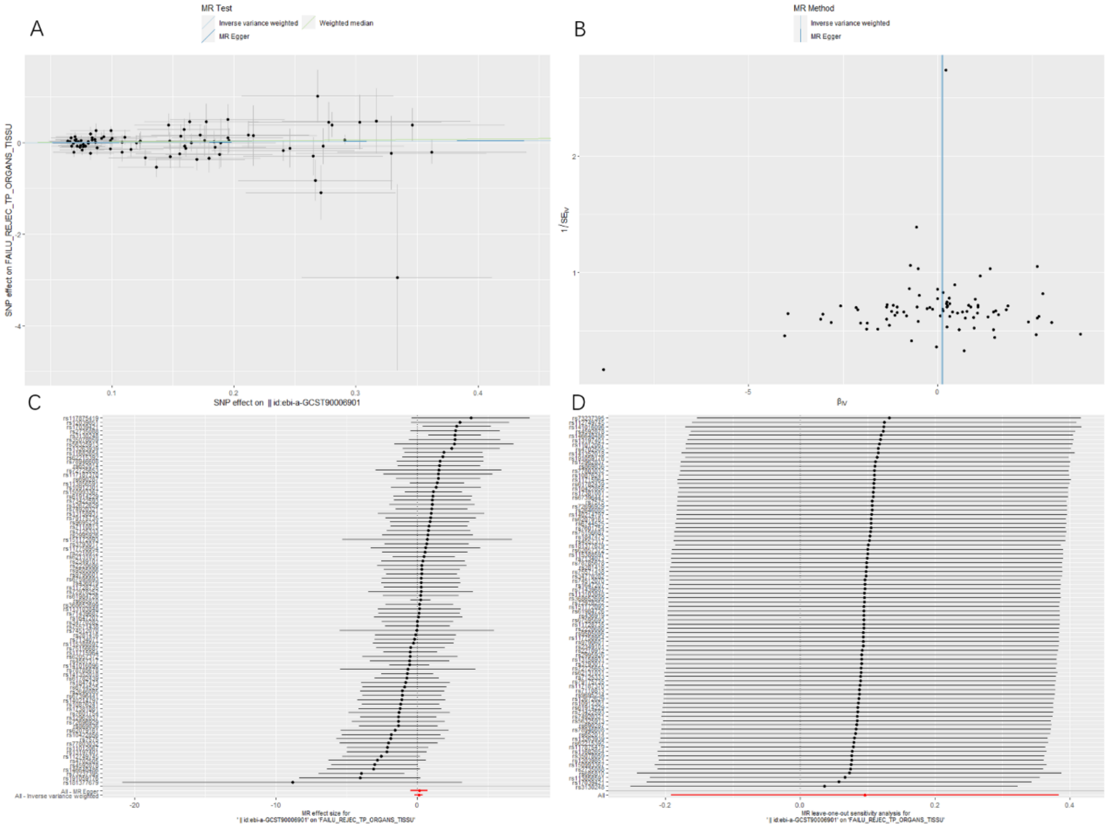

Figure S9. The scatter plot (A), funnel plot (B), forest plot (C) and leave-one-out plot (D) for the association of EBV ZEBRA antibody levels on allograft dysfunction in the forward MR analysis.

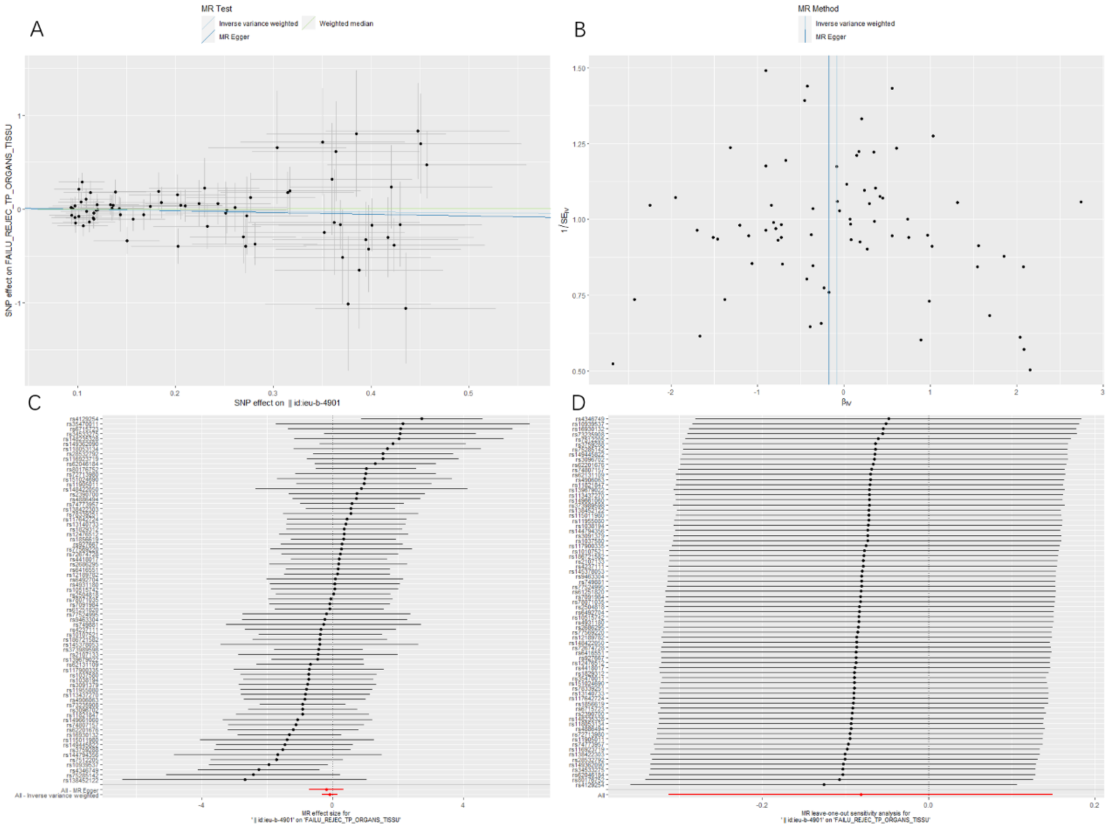

Figure S10. The scatter plot (A), funnel plot (B), forest plot (C) and leave-one-out plot (D) for the association of anti-EBV IgG levels on allograft dysfunction in the forward MR analysis.

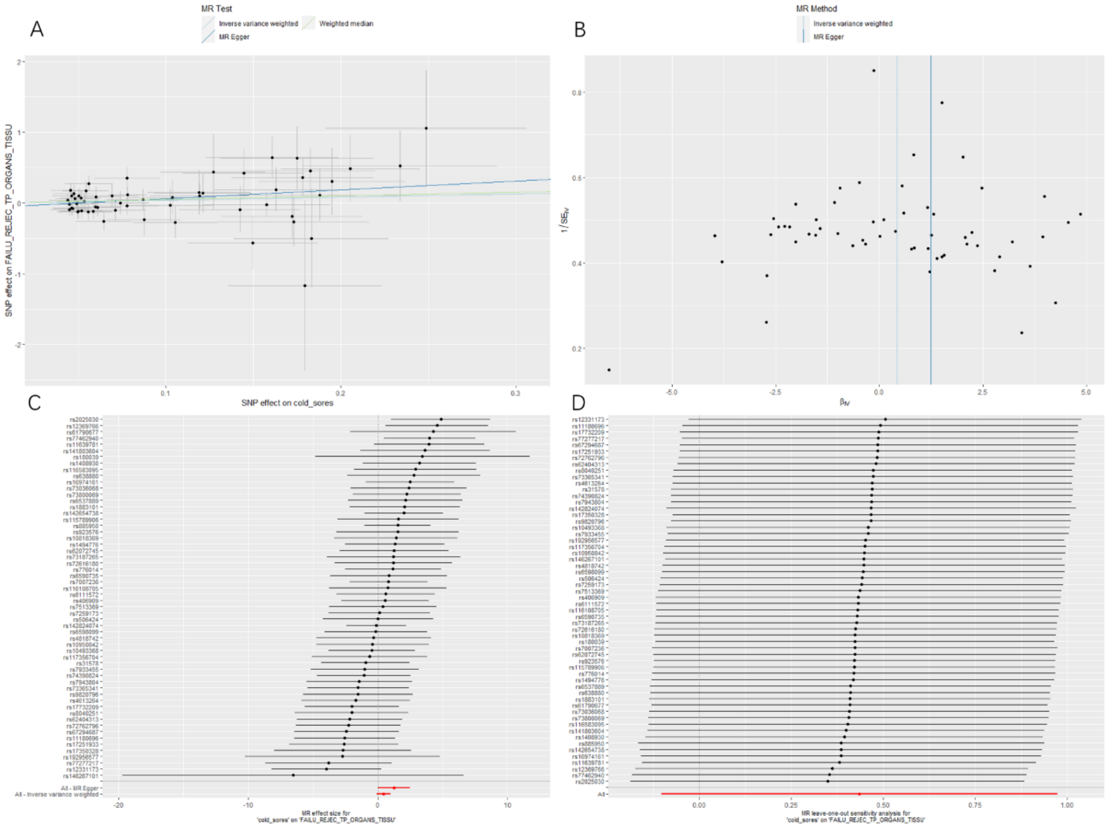

Figure S11. The scatter plot (A), funnel plot (B), forest plot (C) and leave-one-out plot (D) for the association of cold scores on allograft dysfunction in the forward MR analysis.

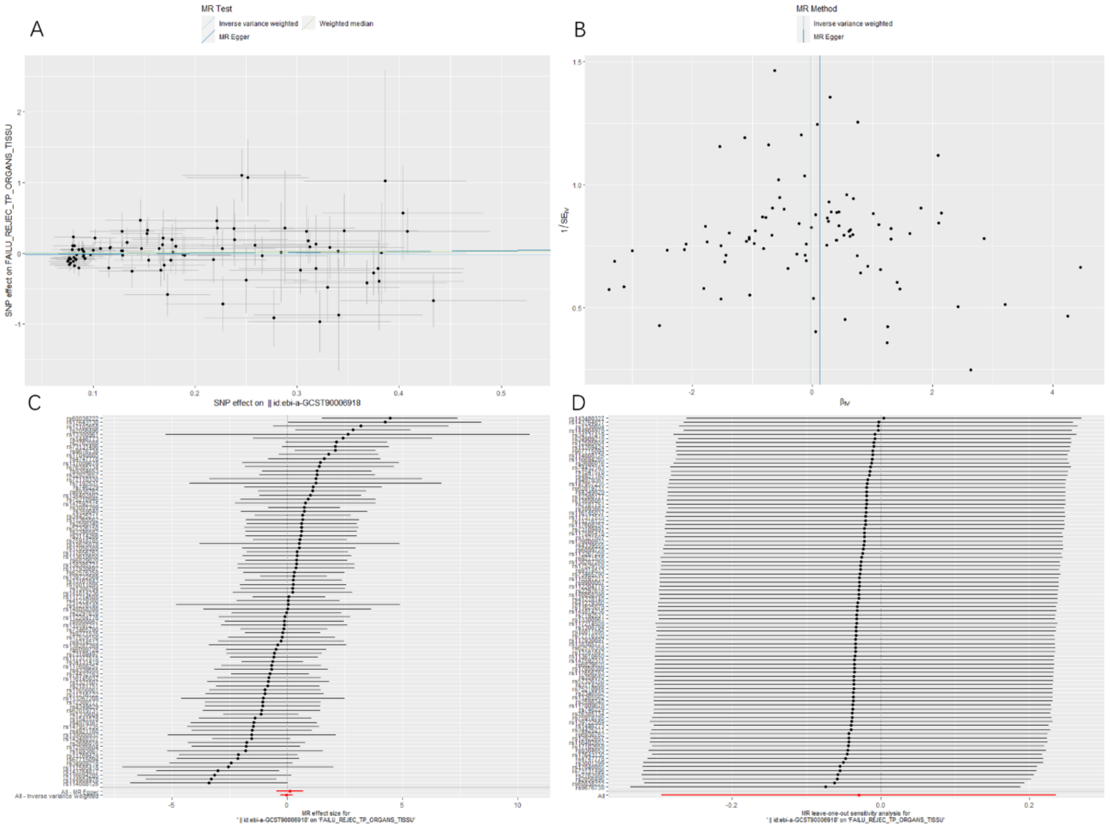

Figure S12. The scatter plot (A), funnel plot (B), forest plot (C) and leave-one-out plot (D) for the association of HSV-1 mgG-1 antibody levels on allograft dysfunction in the forward MR analysis.

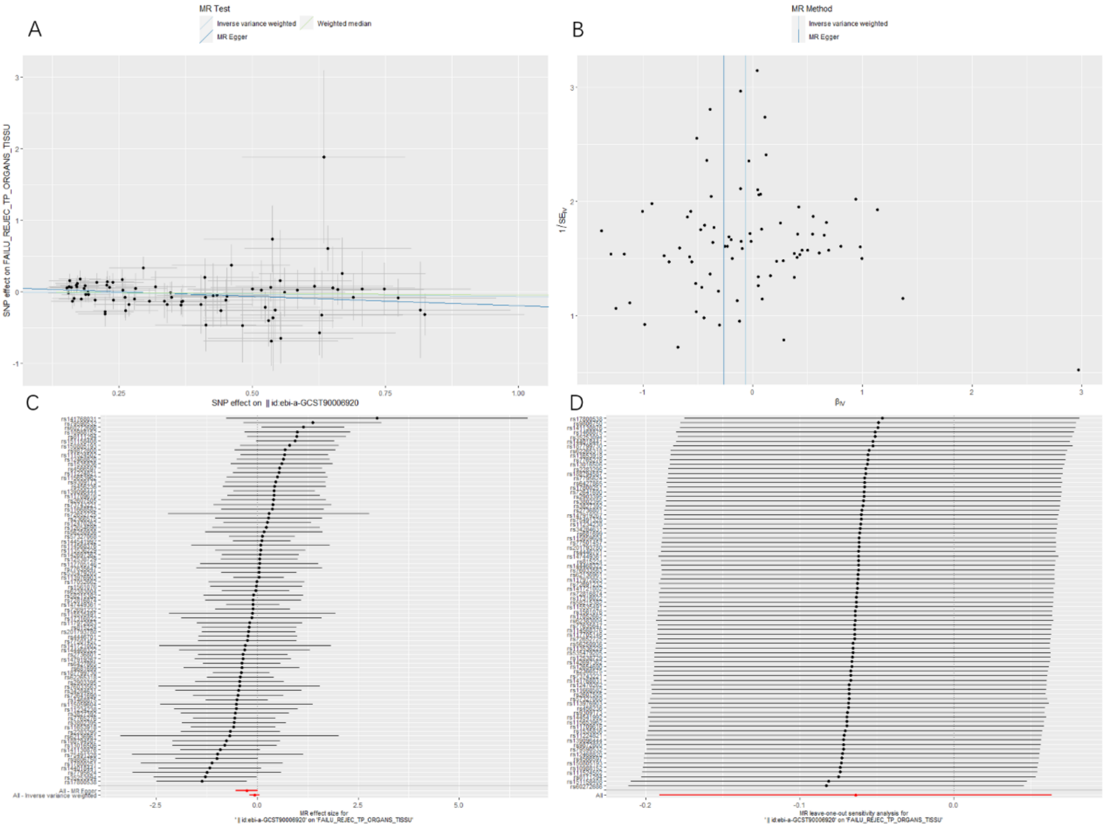

Figure S13. The scatter plot (A), funnel plot (B), forest plot (C) and leave-one-out plot (D) for the association of HSV-2 mgG-1 antibody levels on allograft dysfunction in the forward MR analysis.

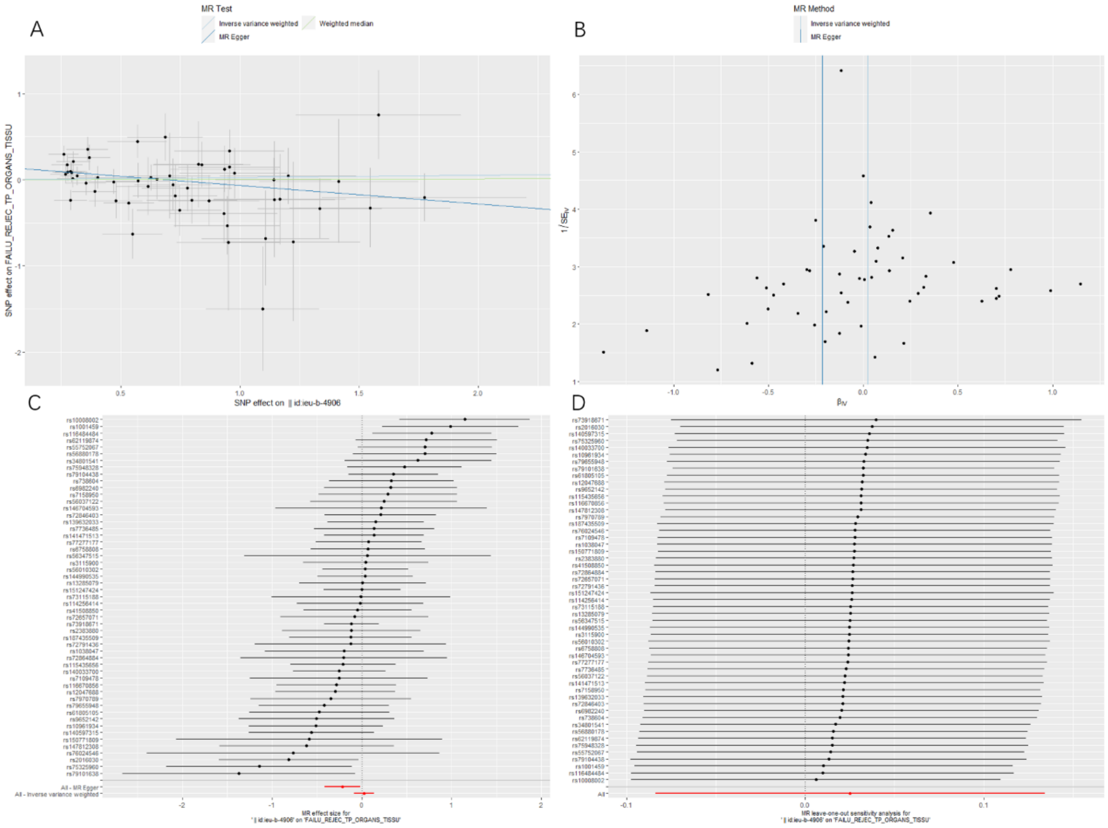

Figure S14. The scatter plot (A), funnel plot (B), forest plot (C) and leave-one-out plot (D) for the association of anti-HSV-1 IgG levels on allograft dysfunction in the forward MR analysis.

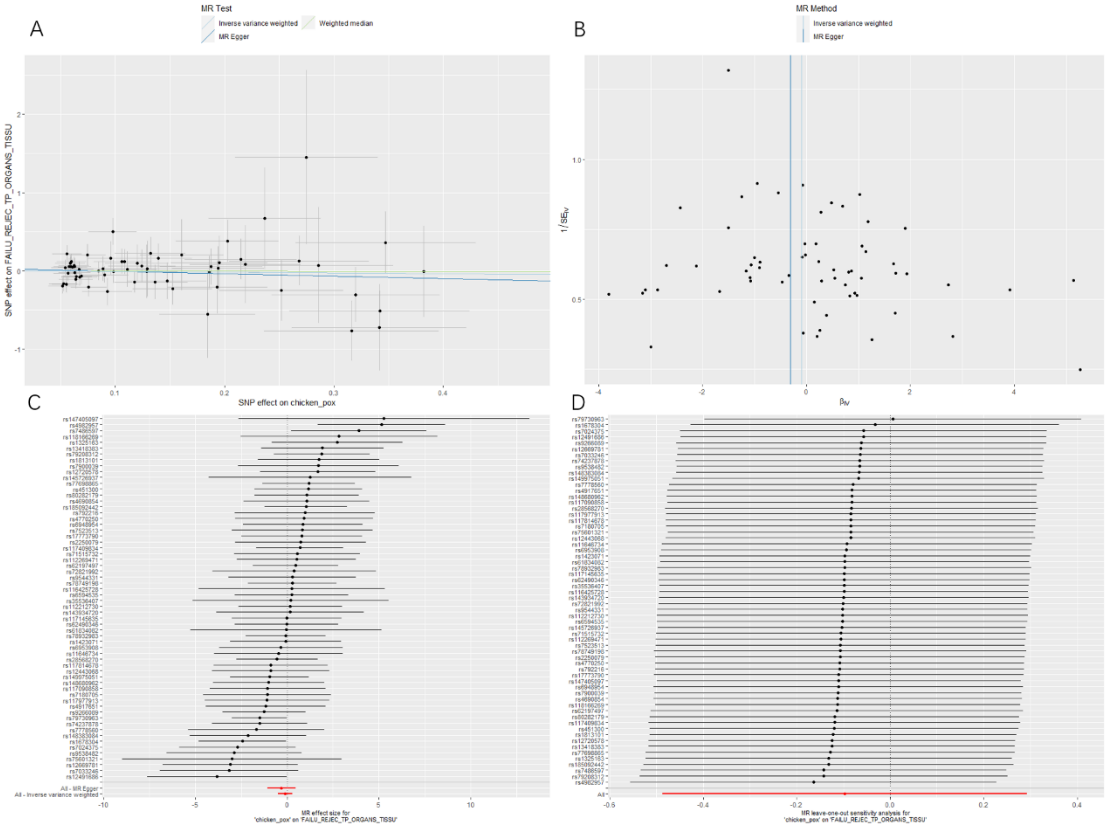

Figure S15. The scatter plot (A), funnel plot (B), forest plot (C) and leave-one-out plot (D) for the association of chickenpox on allograft dysfunction in the forward MR analysis.

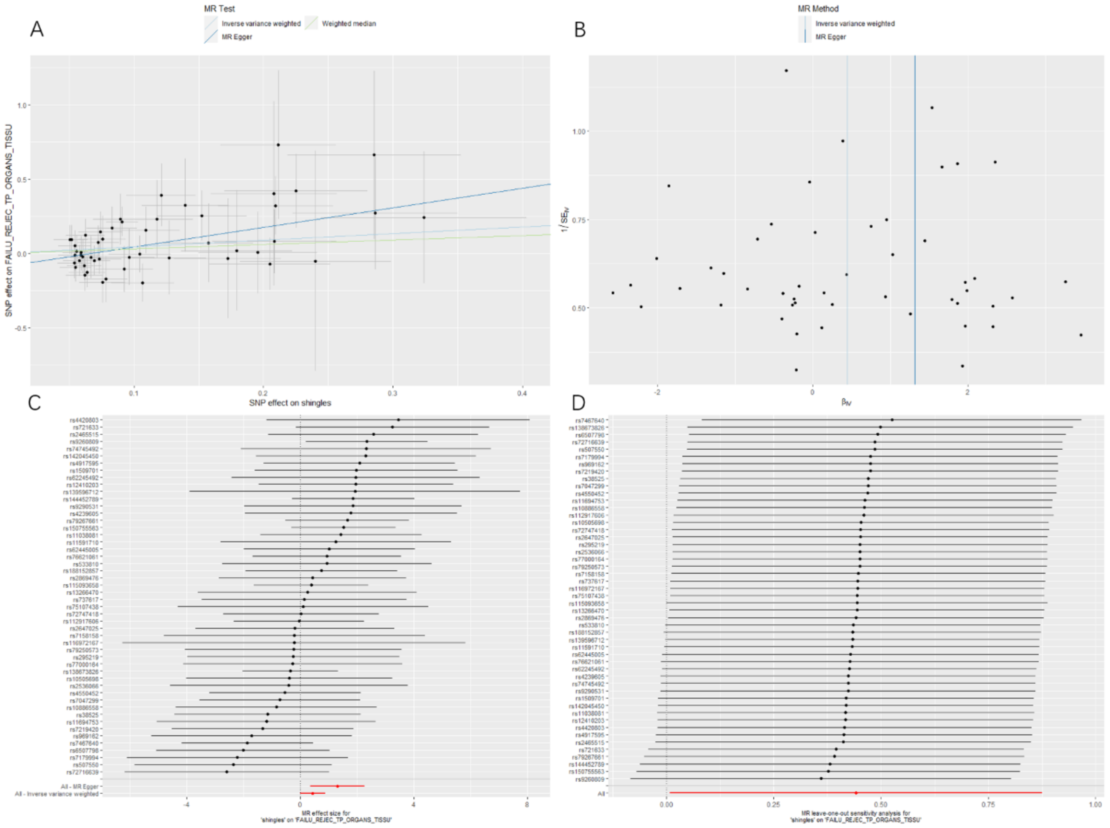

Figure S16. The scatter plot (A), funnel plot (B), forest plot (C) and leave-one-out plot (D) for the association of shingles on allograft dysfunction in the forward MR analysis.

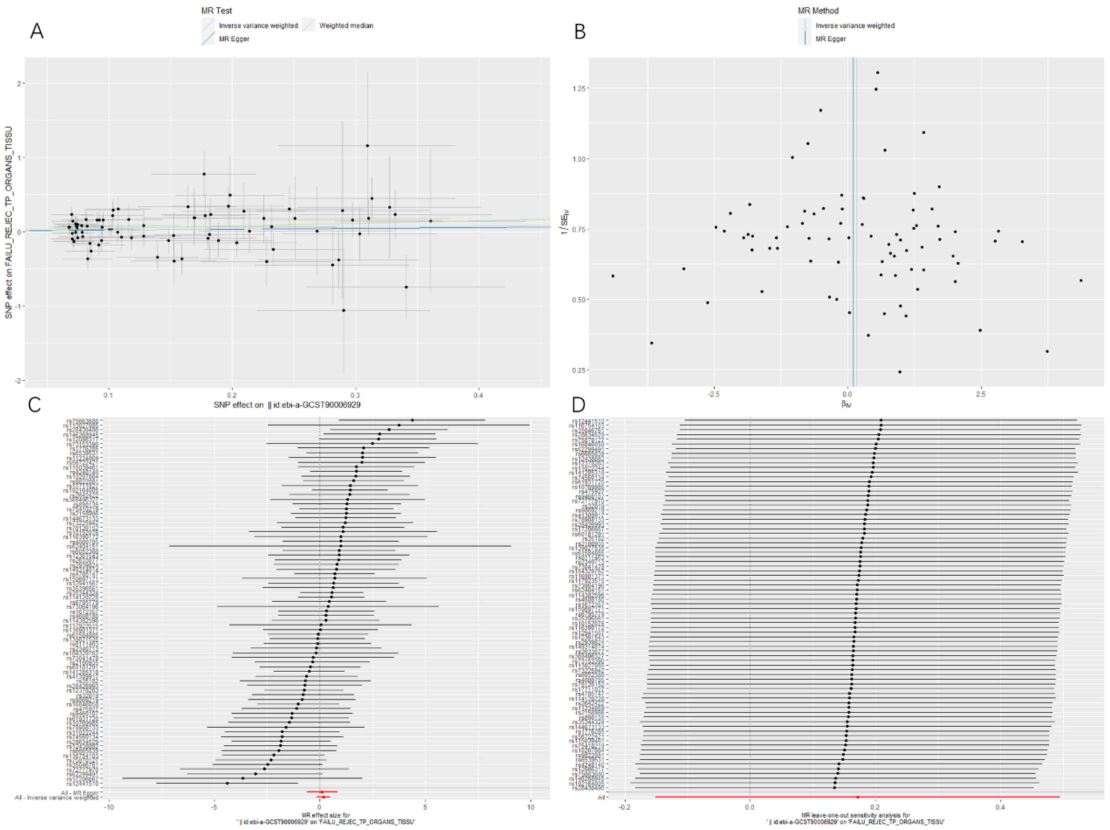

Figure S17. The scatter plot (A), funnel plot (B), forest plot (C) and leave-one-out plot (D) for the association of VZV glycoproteins E and I antibody levels on allograft dysfunction in the forward MR analysis.

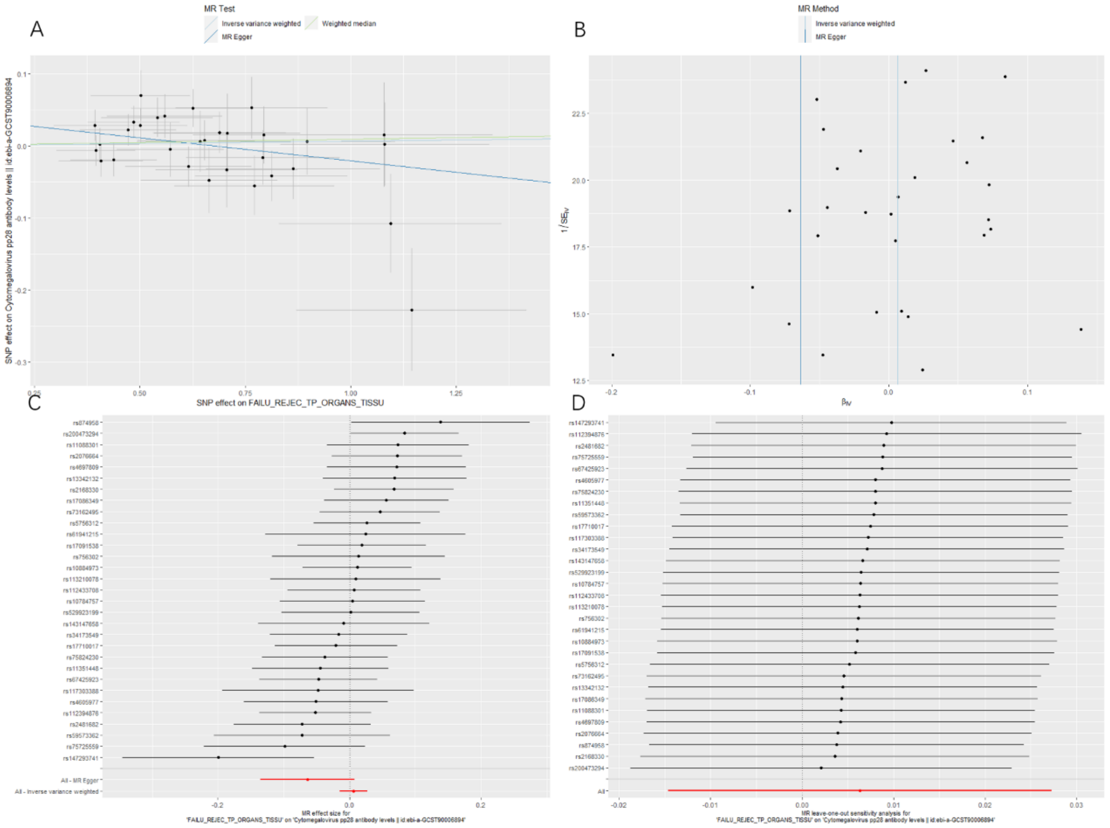

Figure S18. The scatter plot (A), funnel plot (B), forest plot (C) and leave-one-out plot (D) for the association of allograft dysfunction on CMV pp28 antibody levels in the reverse MR analysis.

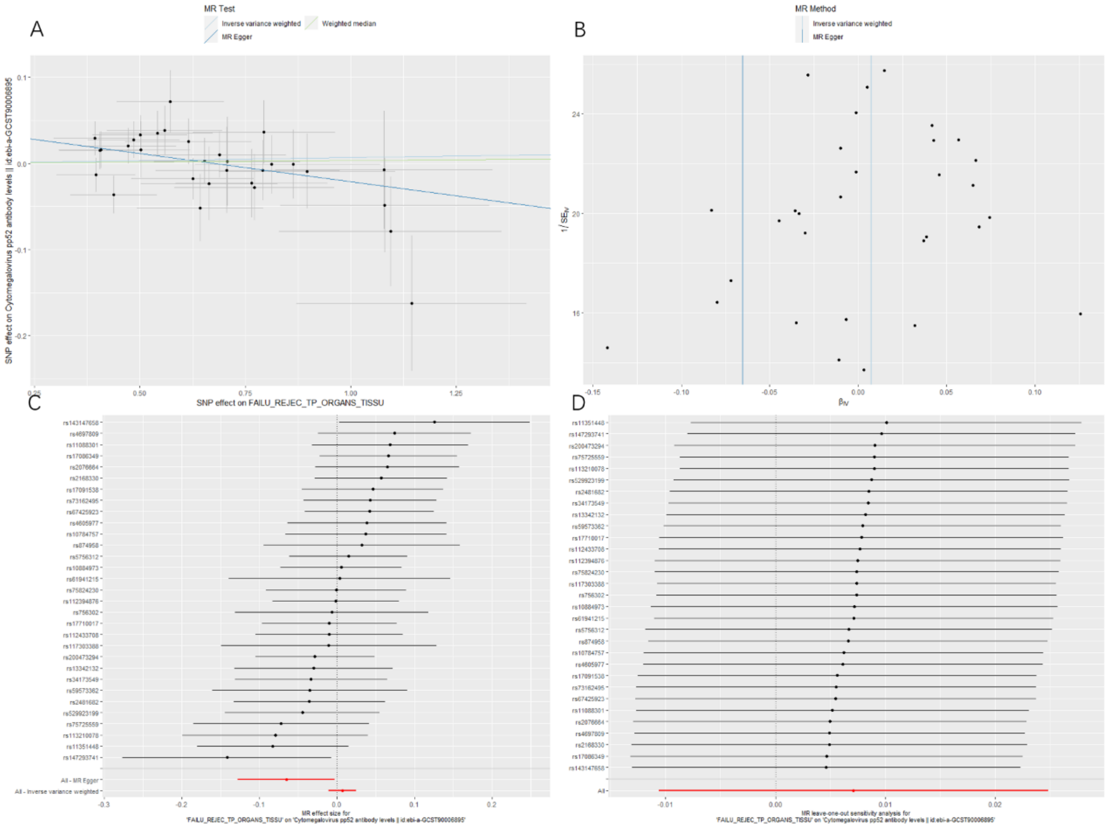

Figure S19. The scatter plot (A), funnel plot (B), forest plot (C) and leave-one-out plot (D) for the association of allograft dysfunction on CMV pp52 antibody levels in the reverse MR analysis.

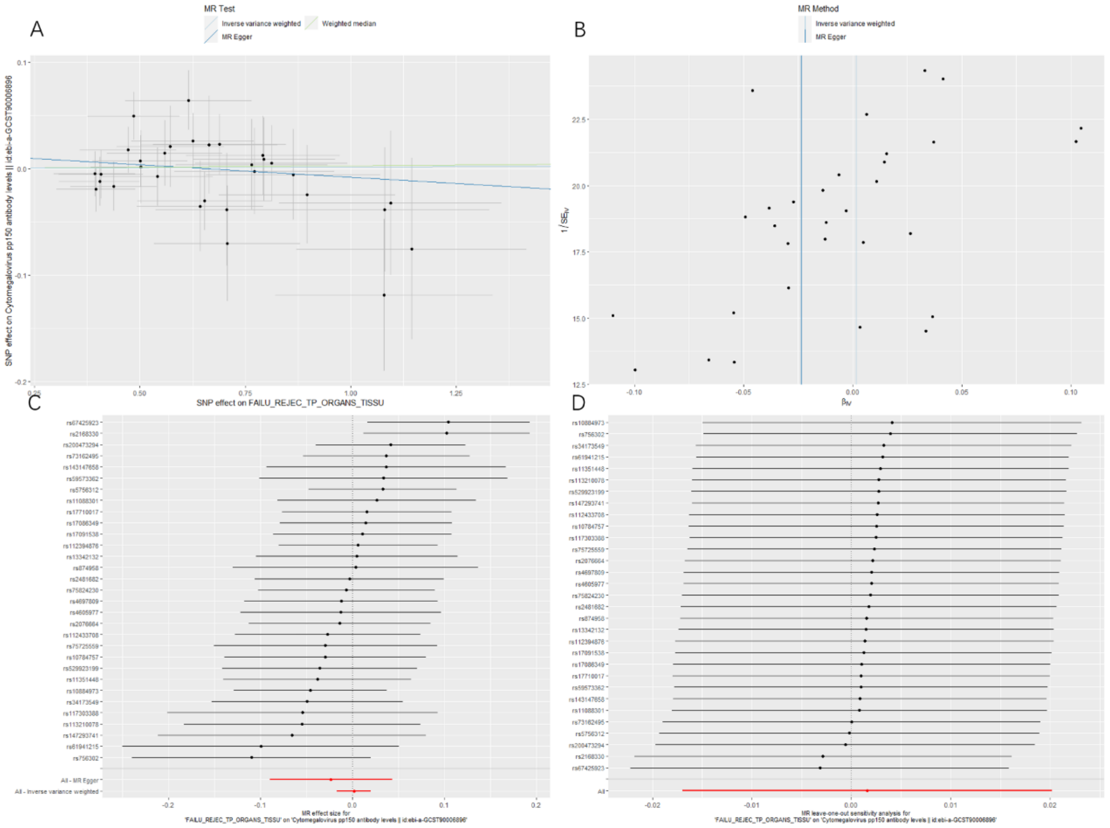

Figure S20. The scatter plot (A), funnel plot (B), forest plot (C) and leave-one-out plot (D) for the association of allograft dysfunction on CMV pp150 antibody levels in the reverse MR analysis.

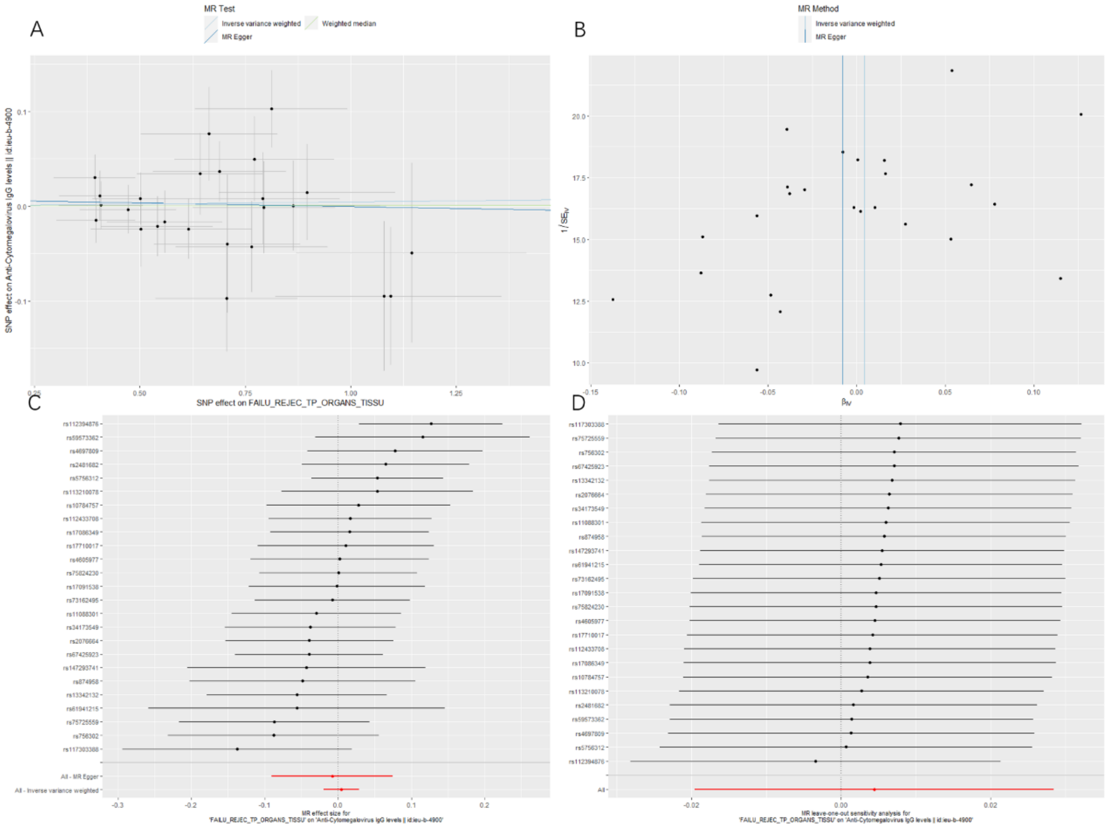

Figure S21. The scatter plot (A), funnel plot (B), forest plot (C) and leave-one-out plot (D) for the association of allograft dysfunction on anti-CMV IgG levels in the reverse MR analysis.

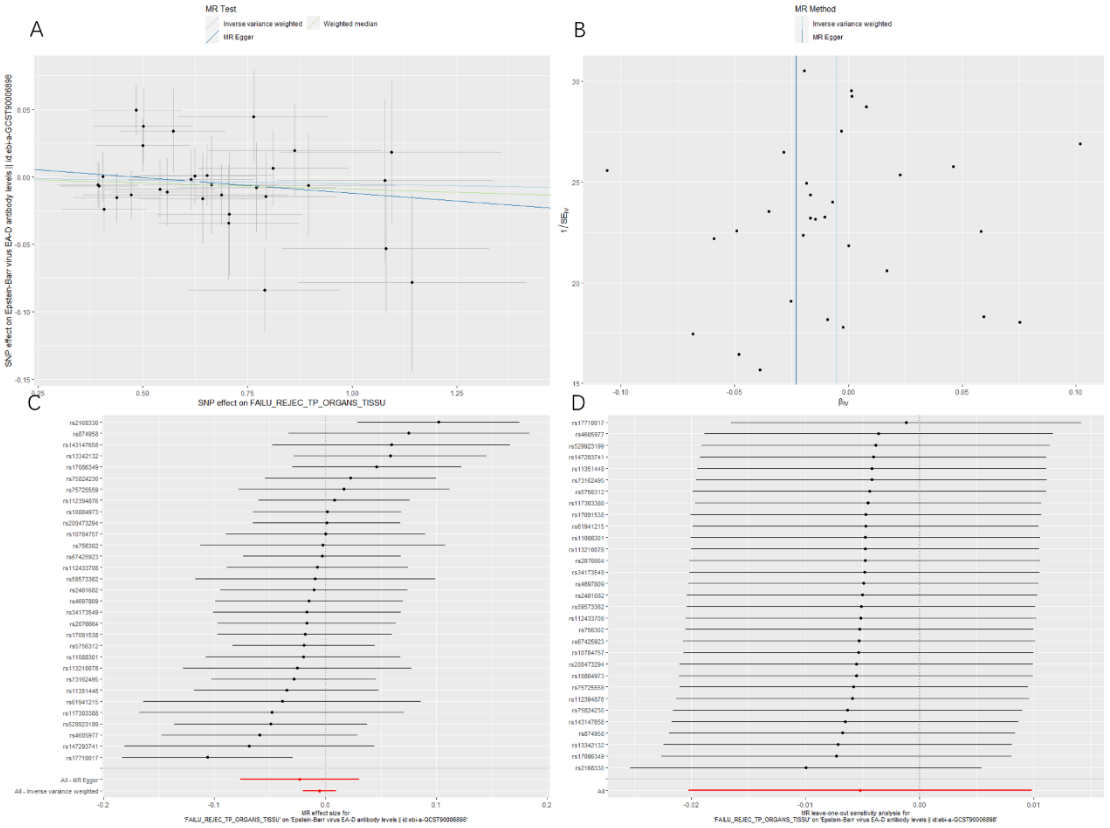

Figure S22. The scatter plot (A), funnel plot (B), forest plot (C) and leave-one-out plot (D) for the association of allograft dysfunction on EBV EA-D antibody levels in the reverse MR analysis.

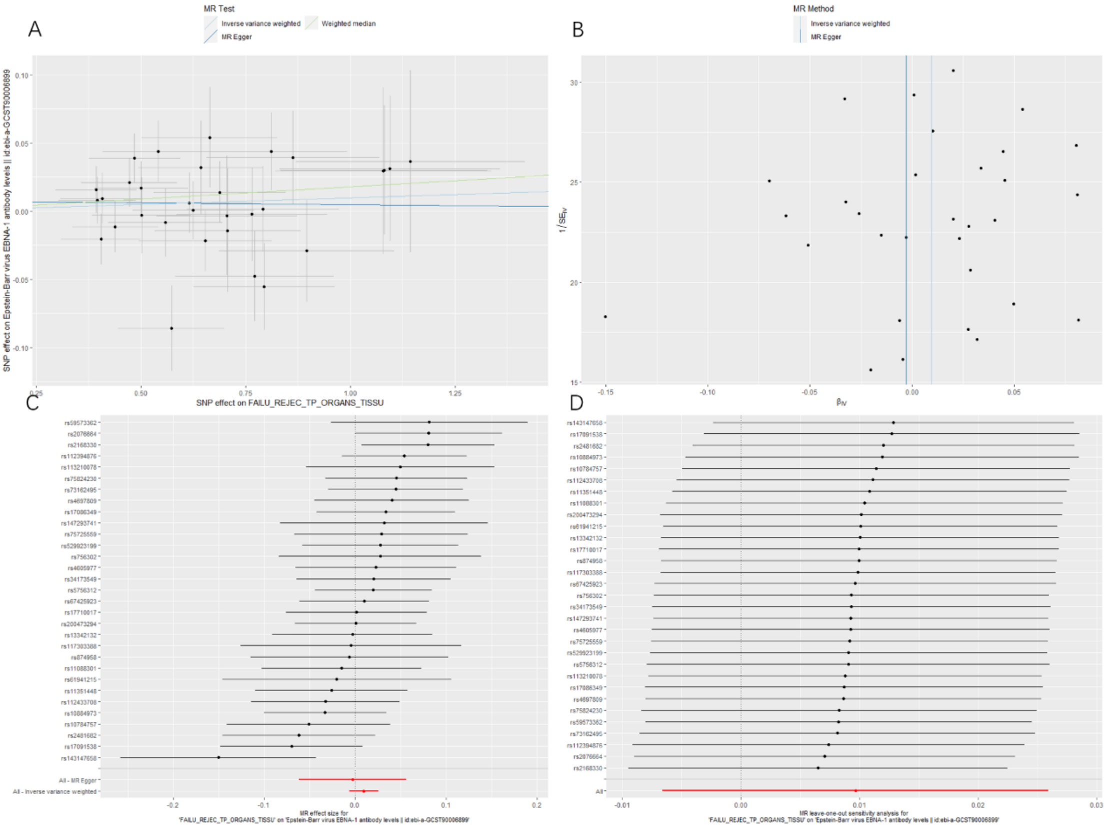

Figure S23. The scatter plot (A), funnel plot (B), forest plot (C) and leave-one-out plot (D) for the association of allograft dysfunction on EBV EBNA-1 antibody levels in the reverse MR analysis.

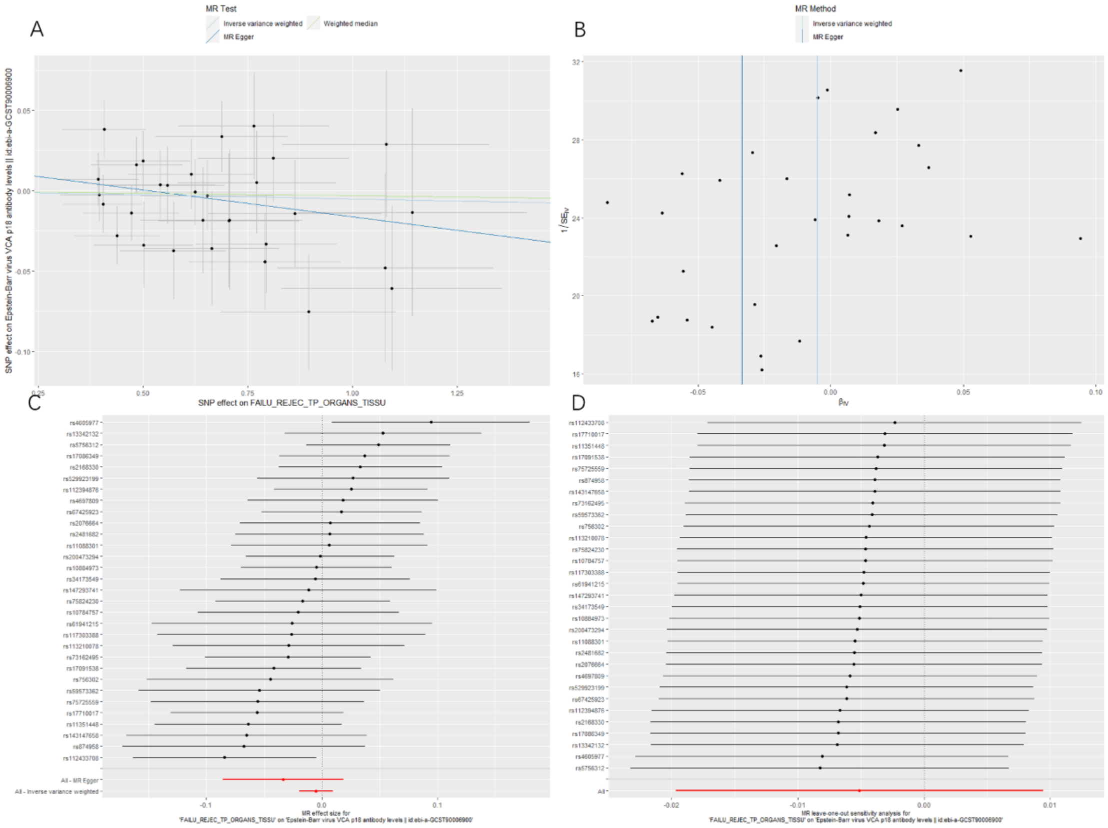

Figure S24. The scatter plot (A), funnel plot (B), forest plot (C) and leave-one-out plot (D) for the association of allograft dysfunction on EBV VCA p18 antibody levels in the reverse MR analysis.

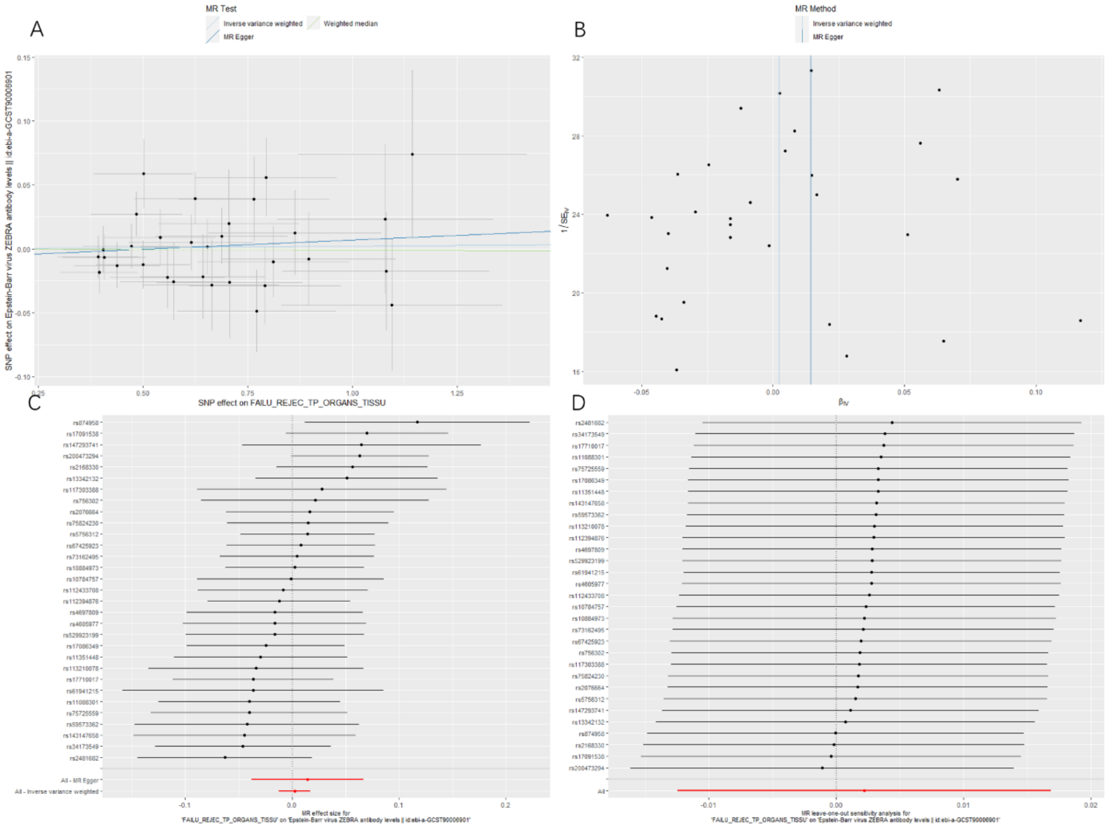

Figure S25. The scatter plot (A), funnel plot (B), forest plot (C) and leave-one-out plot (D) for the association of allograft dysfunction on EBV ZEBRA antibody levels in the reverse MR analysis.

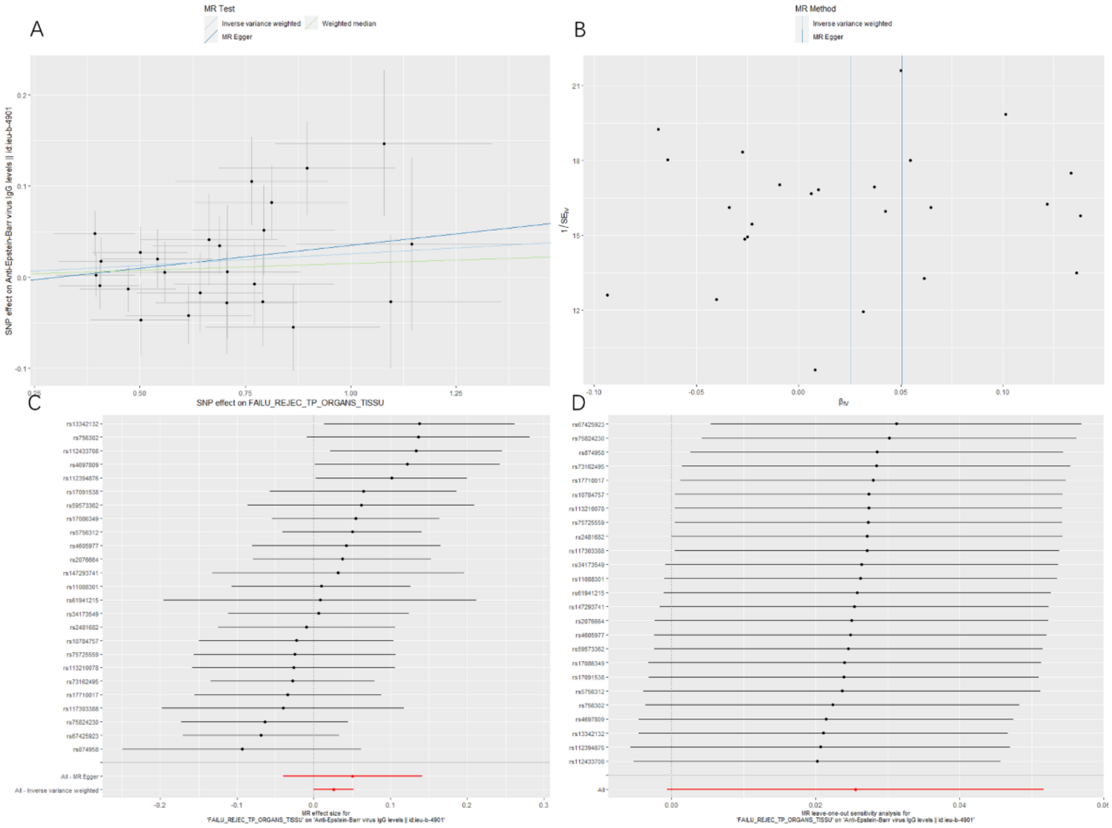

Figure S26. The scatter plot (A), funnel plot (B), forest plot (C) and leave-one-out plot (D) for the association of allograft dysfunction on anti-EBV IgG levels in the reverse MR analysis.

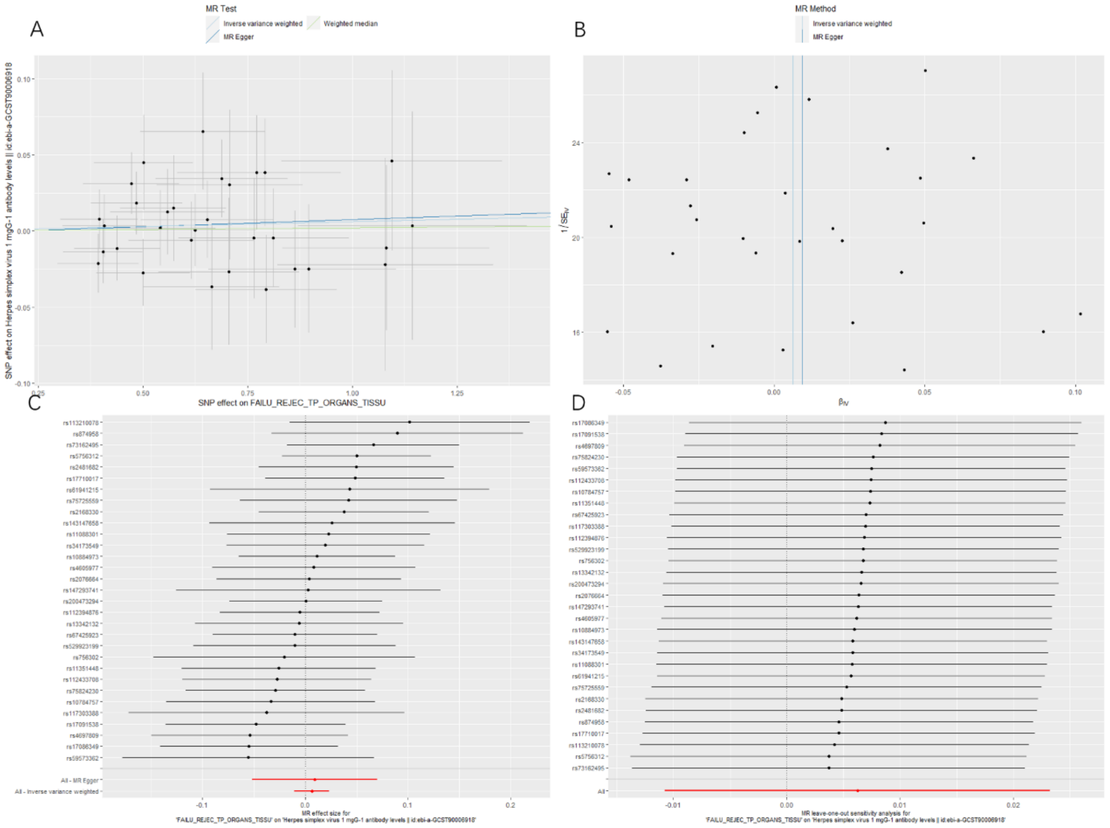

Figure S27. The scatter plot (A), funnel plot (B), forest plot (C) and leave-one-out plot (D) for the association of allograft dysfunction on HSV-1 mgG-1 antibody levels in the reverse MR analysis.

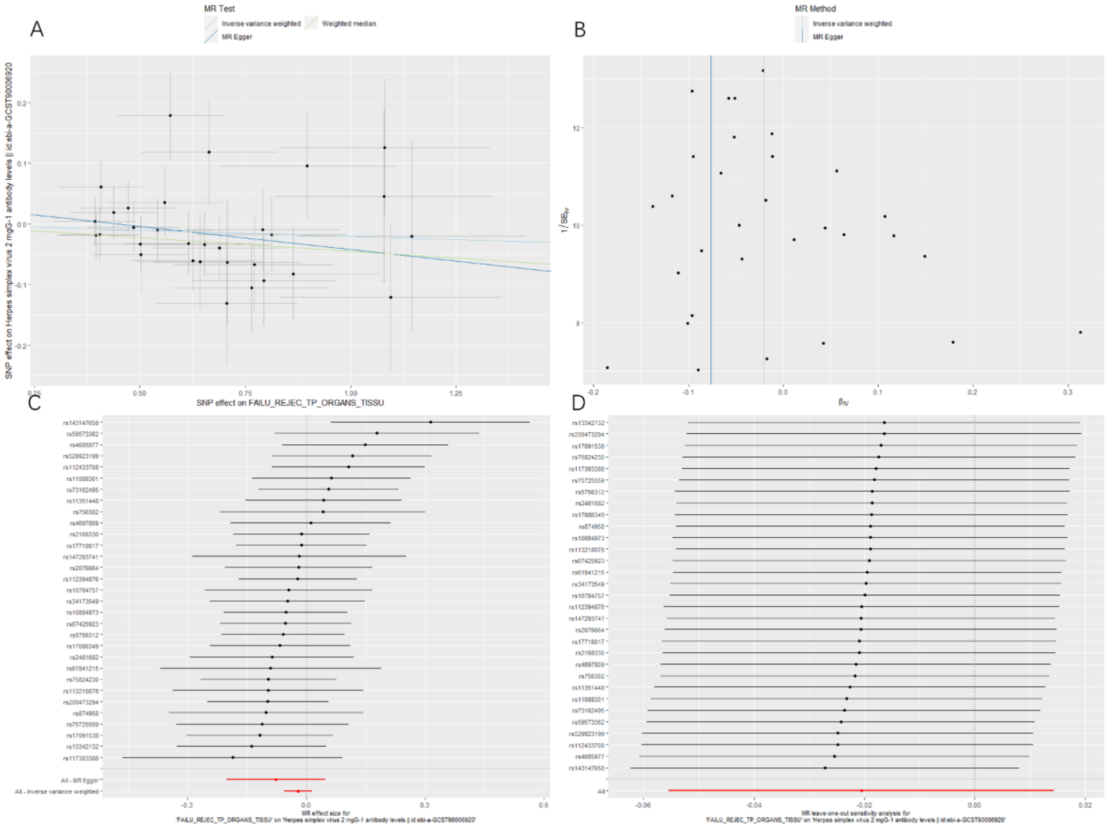

Figure S28. The scatter plot (A), funnel plot (B), forest plot (C) and leave-one-out plot (D) for the association of allograft dysfunction on HSV-2 mgG-1 antibody levels in the reverse MR analysis.

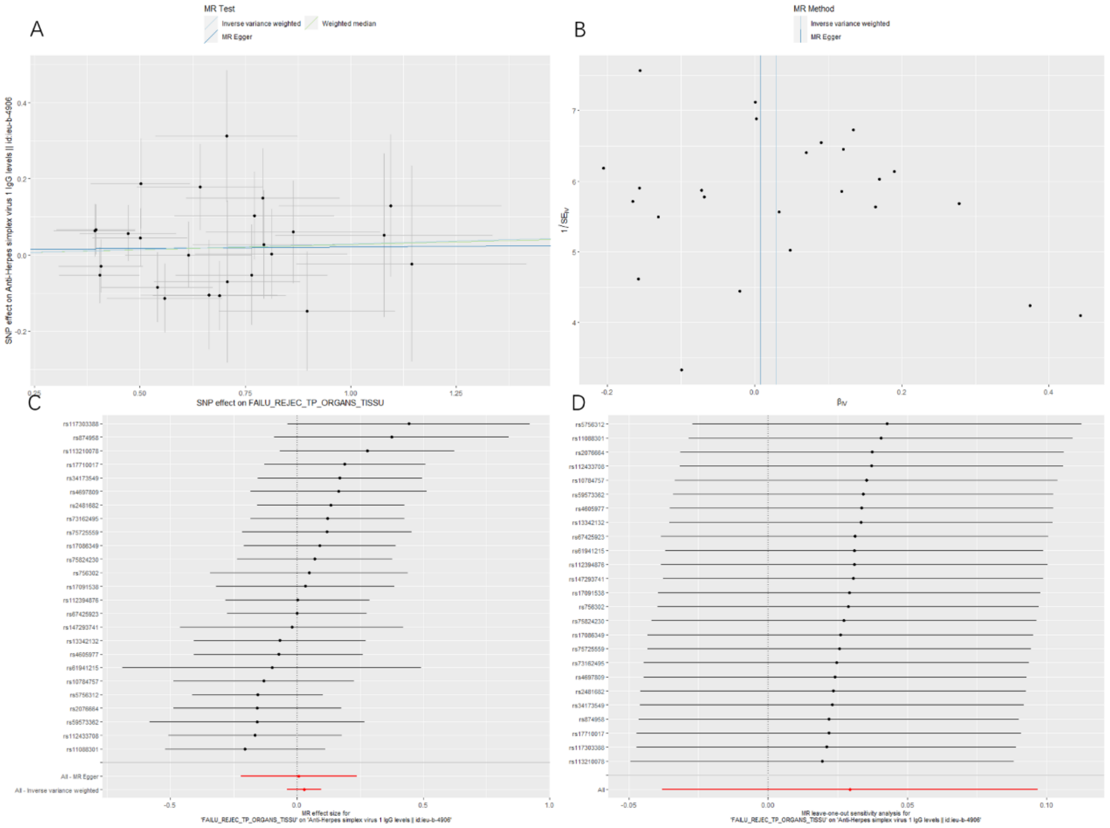

Figure S29. The scatter plot (A), funnel plot (B), forest plot (C) and leave-one-out plot (D) for the association of allograft dysfunction on anti-HSV-1 IgG levels in the reverse MR analysis.

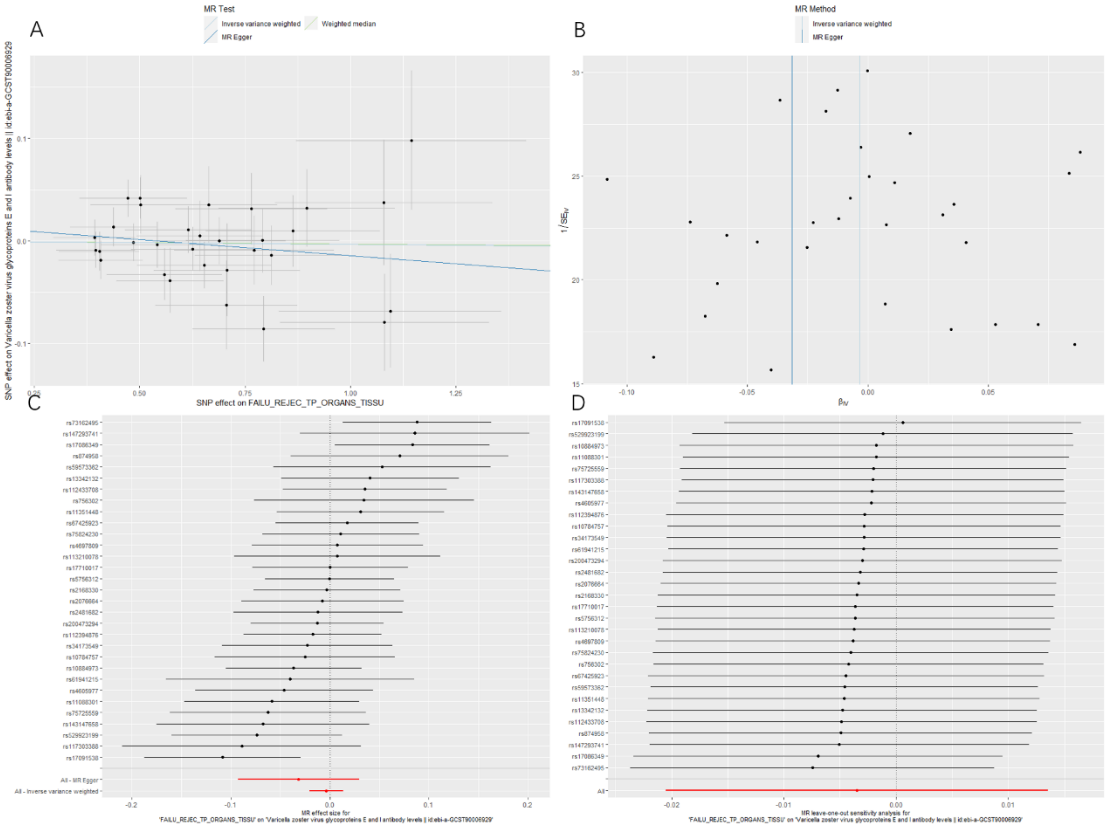

Figure S30. The scatter plot (A), funnel plot (B), forest plot (C) and leave-one-out plot (D) for the association of allograft dysfunction on VZV glycoproteins E and I antibody levels in the reverse MR analysis.
